# Exploring the fate of mRNA in aging seeds: protection, destruction, or slow decay?

**DOI:** 10.1093/jxb/ery215

**Published:** 2018-06-12

**Authors:** Margaret B Fleming, Eric L Patterson, Patrick A Reeves, Christopher M Richards, Todd A Gaines, Christina Walters

**Affiliations:** 1 USDA-ARS, National Laboratory for Genetic Resource Preservation, Fort Collins, CO, USA; 2 Department of Bioagricultural Sciences and Pest Management, Colorado State University, Fort Collins, CO, USA

**Keywords:** Aging, degradation, dry, free radical, *Glycine max*, seeds, storage, transcriptome, whole molecule sequencing

## Abstract

Seeds exist in the vulnerable state of being unable to repair the chemical degradation all organisms suffer, which slowly ages seeds and eventually results in death. Proposed seed aging mechanisms involve all classes of biological molecules, and degradation of total RNA has been detected contemporaneously with viability loss in dry-stored seeds. To identify changes specific to mRNA, we examined the soybean (*Glycine max*) seed transcriptome, using new, whole-molecule sequencing technology. We detected strong evidence of transcript fragmentation in 23-year-old, compared with 2-year-old, seeds. Transcripts were broken non-specifically, and greater fragmentation occurred in longer transcripts, consistent with the proposed mechanism of molecular fission by free radical attack at random bases. Seeds died despite high integrity of short transcripts, indicating that functions encoded by short transcripts are not sufficient to maintain viability. This study provides an approach to probe the asymptomatic phase of seed aging, namely by quantifying transcript degradation as a function of storage time.

## Teaser

Aged soybean seeds show widespread cleavage of long mRNA transcripts with no particular relationship to transcript function, consistent with extant hypotheses of aging mechanisms.

## Introduction

Seeds have a limited lifespan that depends on species, growth conditions, and storage environment. The inevitable death of dry-stored seeds arises from accumulation of molecular damage, particularly non-enzymatic oxidation of proteins, lipids, and nucleic acids (Oracz *et al.*, 2007; Terskikh *et al.*, 2008; Rajjou *et al.*, 2008; Kranner *et al.*, 2011). A decreasing proportion of surviving seeds attests to accumulated damage, but is only apparent after seeds start dying (Walters *et al.*, 2005, 2010). Assessing sub-lethal damage requires a different approach. The inherent fragility of RNA makes it a promising target for quantifying sub-lethal damage, and automated RNA integrity assays, such as the RNA integrity number (RIN), facilitate this approach (Schroeder *et al.*, 2006; Fleming *et al.*, 2017). We found that integrity of total RNA, measured using RIN, within dry-stored seeds is strongly and positively correlated with germination potential (Fleming *et al.*, 2017). RIN mainly reflects integrity of rRNA, and here we explore mRNA as a potentially more sensitive marker of aging.

In dry seeds, mRNA persists, possibly for decades, until germination commences (Dure and Waters, 1965). With the exception of dry seeds, molecules of mRNA are generally short-lived, on the order of hours to days. The usually limited lifespan results from post-transcriptional regulation of mRNA abundance and targeted catalysis of damaged RNA (Shoemaker and Green, 2012; Zhang and Guo, 2017). Cells generally turnover rather than repair transcripts. Degradation of undamaged mRNA is initiated by deadenylation, and followed by decapping and 5′–3′ exonuclease degradation or by 3′–5′ exosomal degradation (Zhang and Guo, 2017). RNA is easily damaged compared with DNA, which is double-stranded, chromatin-bound, and located in the nucleus. The strand backbone of mRNA is vulnerable to truncation and nucleobases can be converted to abasic sites or modified (Wurtmann and Wolin, 2010). Decay mechanisms include ‘nonsense-mediated’, when there is a premature stop codon, and ‘no-go’ and ‘non-stop’ when there are problems during translation (Shoemaker and Green, 2012). Transcripts are usually endonucleolytically cleaved, and the resulting fragments are degraded from the cleavage site outward, in the 5′–3′ direction by exonuclease Xrn1 and in the 3′–5′ direction by the cytoplasmic exosome (Shoemaker and Green, 2012).

There is extensive evidence that seeds translate stored mRNA during germination using stored ribosomes (Basbouss-Serhal *et al.*, 2015; Bai *et al.*, 2017, and references therein). Whether stored mRNA is sufficient to allow radicle emergence may be species dependent, shown by varying results after treatment with transcriptional inhibitors (Jendrisak, 1980; Rajjou *et al.*, 2004; Sano *et al.*, 2012). Chemical damage to stored mRNA, rRNA, and DNA compromises germination progress (Rajjou *et al*, 2012). Shortened telomeres as well as fragmentation, particularly laddering, have been observed during aging (Boubriak *et al.*, 2007; Kranner *et al.*, 2011). Several assays for DNA integrity, such as the comet and TUNEL assays, successfully identify aging damage in seeds (El-Maarouf-Bouteau *et al.*, 2011; Waterworth *et al.*, 2015; Burrieza *et al.*, 2016). The greater stability of DNA makes it a less promising molecule to detect changes during asymptomatic seed aging.

Mature, dry seeds often serve as the final time point in transcriptomic studies of seed development (Jones *et al.*, 2010; Pereira Lima *et al.*, 2017) or the initial time point in seed germination series (Nakabayashi *et al.*, 2005; Bellieny-Rabelo *et al.*, 2016; Bai *et al.*, 2017). Analyses of transcriptome changes during seed storage are rare and focused on dormancy relief in dry after-ripened or hydrated seeds (Johnson and Dyer, 2000; Bazin *et al.*, 2011; Gao *et al.*, 2013; El-Maarouf-Bouteau *et al.*, 2013; Cao *et al.*, 2016) or following artificial aging of seeds exposed for a short time to warm, wet conditions (Chen *et al.*, 2013). Both physiological transitions presume degradation of specific transcript targets. The mRNA within dry seeds can sustain germination completion in several species (Rajjou *et al.*, 2004; Nakabayashi *et al.*, 2005; Kimura and Nambara, 2010; Sano *et al.*, 2012) and so there may be a requirement to protect certain transcripts.

We evaluate changes in mRNA in dry soybean seeds. Seeds harvested in 1994 were detectably aging in 2016, while seeds harvested in 2015 were completely viable, and integrity of total RNA differed in the two samples (Fleming *et al.*, 2017). Our work here focuses on the mRNA fraction and uses third-generation sequencing technology to sequence whole molecules without prior fragmentation (Oikonomopoulos *et al.*, 2016), so that full-length and fragmented transcripts from the two cohorts could be distinguished and quantified. We asked whether degradation patterns appeared random or targeted in a dry seed by determining if particular gene functions were preserved or sacrificed and if particular sequence motifs or cleavage sites were more or less prone to degradation. As a practical goal, we sought candidate transcripts to serve as diagnostic markers of the aging process.

## Materials and methods

### Plant material

Soybean (*Glycine max,* cv. ‘Williams 82’) seeds were part of a legacy collection described earlier (Fleming *et al.*, 2017). Seeds were stored at 5 °C and approximately 35% relative humidity from 3–6 months after harvest until use. We compared transcriptomes of embryonic axes from seeds harvested in 1994 and 2015 (1994H and 2015H, respectively) and quantified RNA oxidation products from embryonic axes from seeds harvested in 1989, 1995, 1999, and 2015 (1989H, 1995H, 1999H, and 2015H).

### Viability monitoring

Germination assays were conducted on 28 January 2016 and 2 June 2017 (Fleming *et al.*, 2017). Seeds were rolled in moist germination paper (Anchor, St Paul, MN, USA) (three rolls of 15–17 seeds each), incubated in the dark at 25 °C for 7 d and then assessed for radicle emergence. The 1994H cohort was in the midst of rapid mortality, evident from 60% germination in the 2016 assay. Germination ranged from 0 to 90% for seed lots harvested in 1989, 1995, and 1999, used for RNA oxidation assessment. All 2015H seeds germinated normally.

### RNA extraction and characterization

RNA was extracted for oxidation analysis from five replicate pools of six dry embryonic axes each from the 1989H, 1995H, 1999H, and 2015H cohorts on 23 January 2017. RNA was extracted for sequencing from five individual dry embryonic axes from the 1994H and 2015H cohorts on 1 March 2017. Axes were ground with a no. 40 BB steel shot in a Retsch (Haan, Germany) Bead Mill at 30 oscillations s^−1^. Axes from single seeds were ground for 1 min under liquid nitrogen in microcentrifuge tubes containing 1 mg of polyvinylpyrrolidone-40 (Fisher Scientific, Fair Lawn, NJ, USA). Samples for oxidation analysis (a pool of six axes) were ground for 4 min at 22 °C, in 450 µl of Buffer RLC (Qiagen, Hilden, Germany) including 1 mg of polyvinylpyrrolidone-40 per 10 mg tissue, 10 µl ml^−1^ of β-mercaptoethanol, 2.5 mM deferoxamine mesylate (Sigma-Aldrich, St Louis, MO, USA), and 0.5 mM butylated hydroxytoluene (Sigma-Aldrich). After grinding, the Qiagen Plant RNEasy kit was used for all samples, following the recommended protocol modified by repeating the final wash with 500 µl of buffer RPE to reduce guanidine hydrochloride carry-over.

RNA yield was quantified using a Nanodrop 1000 spectrophotometer (Thermo Fisher Scientific, Wilmington, DE, USA). Samples were diluted to 1 ng µl^−1^ in nuclease-free water. Integrity of diluted RNA was quantified on an Agilent (Waldbronn, Germany) Bioanalyzer, using Agilent RNA 6000 Pico chips and the Plant RNA Pico or mRNA Pico Series II assay (Agilent 2100 Expert software version B.02.08.SI648 R3), following the manufacturer’s protocols.

### mRNA purification

DNA was removed from total RNA extracted from 2015H and 1994H single axes, using 26 µg for each sample, with the Turbo DNA-*free* kit (Thermo Fisher Scientific, Waltham, MA, USA), following the manufacturer’s ‘rigorous’ protocol. The NEBNext Poly(A) mRNA Magnetic Isolation Module (New England Biolabs, Ipswich, MA, USA) was used to enrich for mRNA according to the manufacturer’s protocol.

### cDNA synthesis

Synthesis of cDNA for sequencing followed the strand-switching protocol from Oxford Nanopore Technologies (Supplementary Methods S1 at *JXB* online). Using this protocol, an incomplete cDNA sequence should arise from an incomplete or fragmented template (Zhu *et al.*, 2001).

For qPCR, cDNA libraries were synthesized from three 1994H and three 2015H single-axis RNA extractions, using oligo(dT) or random hexamer primers with both poly(A)-selected and total RNA. All reagents were supplied by Invitrogen (Carlsbad, CA, USA). Reverse transcription used 1.6 µg of total RNA (from initial DNase-treated RNA) or 10–20 ng of poly(A)-selected RNA (from samples used for sequencing), 1 µl of 10 mM dNTPs, and either 1 µl of 50 µM oligo(dT)_20_ primer or 50 ng of random hexamer primers in 13 µl. After incubating at 65 °C for 5 min, reactions were snap-cooled in a pre-chilled metal tube rack for 1 min. To the reaction tube, 4 µl of 5× RT buffer, 1 µl of 100 mM DTT, 40 U of RNase OUT, and 200 U of SuperScript® IV reverse transcriptase were added. Reactions were incubated at 23 °C for 10 min, 50 °C for 10 min, and 80 °C for 10 min. RNA was removed by treatment with 2 U of *E. coli* RNase H followed by incubation at 37 °C for 20 min. All cDNA was stored at −18 °C until use.

### Library preparation and MinION sequencing

Libraries were barcoded, pooled, and prepared for sequencing according to Oxford Nanopore protocols (Supplementary Methods S1). Each library pool consisted of five samples: the first pool included two 1994H and three 2015H samples, and the second pool included three 1994H and two 2015H samples. Each library was sequenced on a MinION SpotON Flow Cell MK I (R9.4) (Oxford Nanopore Technologies, Oxford, UK). Sequencing was initiated with the sequencing script ‘NC_48Hr_Sequencing_Run_FLO-MIN106_SQK-LSK108’ in MinKNOW 1.4.2.

### MinION sequence data processing: basecalling, demultiplexing, alignment, and filtering

Sequencing data were basecalled using Albacore 0.8.4 (Oxford Nanopore Technologies). Reads were demultiplexed by barcode using porechop 0.2.0 (https://github.com/rrwick/Porechop, released 3/27/2017) with default settings. Reads were aligned for each sample to *Glycine max_275_Wm82.a2.v1* primary transcripts (Schmutz *et al.*, 2010, available from phytozome.jgi.doe.gov) using the Burrows–Wheeler Aligner 0.7.15 (http://bio-bwa.sourceforge.net/, released 6/1/2016) with the ‘ONT-2D’ option for long reads (Li and Durbin, 2009). BBMap pileup (37.28, https://sourceforge.net/projects/bbmap/, released 6/1/2017) was used to compare aligned and reference transcript lengths, identify transcripts with at least one sequence alignment in all samples, and calculate GC content (%). The number of sequence reads at each base pair was calculated using SAMtools depth (1.4, http://www.htslib.org/, released 13 March 2017). Due to limitations of Albacore 0.8.4, read depth varies across a transcript, especially in homopolymer stretches >5 bp, even when the complete molecule has been deeply sequenced. Sequence data from the External RNA Controls Consortium (ERCC) RNA Spike-In Mix (Invitrogen) showed that read depth varied by transcript abundance and that all 10 libraries were equally sequenced (see Supplementary Fig. S1).

### Transcript analysis: read depth, relative integrity calculations, and GO analysis

For each transcript, read depth was normalized between 0 and 100 and plotted against base pair position to establish the distribution of sequence lengths. The differences between the maximum possible read depth (100) and average 2015H read depth for each base pair position were summed. This value represents transcript degradation in 2015H samples relative to a perfectly intact transcript (relative degradation, RD_2015_). Small and large values indicate high and low transcript integrity, respectively, in 2015H samples. The differences between average 2015H and 1994H read depths at each base pair position were summed. This value represents transcript degradation in 1994H samples relative to 2015H samples (RD_Δ_). Values for RD_2015_ and RD_Δ_ were normalized relative to a perfectly intact transcript with the maximum possible read depth at each base pair position, i.e. transcript length×100. Only bases between 25% and 75% of the transcript length were used for RD calculations because transcript ends frequently had unreliable depth measurements due to poor sequencing or alignment.

To analyze gene functions, we used the PANTHER Overrepresentation Test (www.geneontology.org, released 13 April 2017) with the Gene Ontology (GO) database (released 26 September 2017).

### Quantification of 8-oxo-guanosine: LC-MS/MS

RNA nucleosides guanosine and 8-oxo-guanosine were quantified using LC-MS/MS. RNA from six pooled axes (50–75 µg) was digested to nucleosides in a 200 µl overnight reaction at 37 °C with 200 U of S1 nuclease (Thermo Fisher Scientific), 2.5 mM deferoxamine mesylate and 0.5 mM butylated hydroxytoluene added to the manufacturer’s reaction buffer (pH 4.5). This was followed by addition of 10 U of recombinant shrimp alkaline phosphatase (New England Biolabs), 22 µl of 10× CutSmart Buffer, and 22 µl of 1 M Tris-acetate pH 7.9, and the reaction incubated 1 h at 37 °C in the dark. Enzymes were removed by microfiltration (10 kDa, Amicon Ultra, Sigma-Aldrich). LC-MS/MS was performed on a Waters (Milford, MA, USA) Acquity UPLC coupled to a Waters Xevo TQ-S triple quadrupole mass spectrometer. Chromatographic separations were performed on a Waters T3 stationary phase (1 mm×100 mm, 1.7 μM) column. Mobile phases were methanol (B) and water with 0.1% formic acid as modifier (A). The analytical gradient was: 0 min, 0.1% B; 3.0 min, 0.1% B; 12 min, 55% B; 12.01 min, 97% B; 13 min, 97% B; 13.01 min, 0.1% B; 20 min, 0.1% B. Flow rate was 100 μl min^−1^ and injection volume was 1 μl. Samples were injected directly to measure 8-oxo-guanosine or as a 1:50 dilution in nuclease-free water to measure guanosine. Samples were held at 4 °C in the autosampler, and the column was operated at 45 °C. The MS was operated in selected reaction monitoring mode. Product ions, collision energies, and cone voltages were optimized for each analyte by direct injection of synthetic standards. Inter-channel delay was 3 ms. The MS was operated in positive ionization modes with capillary voltage at 3.2 kV. Source temperature was 150 °C and desolvation temperature 500 °C. Desolvation gas flow was 1000 liters h^−1^, cone gas flow 150 liters h^−1^, and collision gas flow 0.2 ml min^−1^. Nebulizer pressure (nitrogen) was 7 bar. The collision gas was argon. Quantification used a calibration curve generated with authentic standards for both compounds and their corresponding stable-isotope-labeled internal standards in neat solution.

### qPCR

To validate transcript deterioration in 1994H compared with 2015H samples, three transcripts from region A (intact in both cohorts) and three from region B (intact in 2015H samples and degraded in 1994H samples) were chosen for quantitative PCR testing using cDNA. The six transcripts were of similar length (1000–2000 bp). For each transcript, we compared the abundance of a 5′ end amplicon and a 3′ end amplicon (Supplementary Tables S1, S2; and see Results, Supplementary Figs S5, S6). The regions amplified were within the bounds of MinION sequence data. Primers and amplicons had similar length, annealing temperature, and GC content (Supplementary Tables S2, S3). BLAST searches against the non-redundant nucleotide database (NCBI) confirmed that primer pairs targeted a single transcript.

For qPCR, reactions contained 2 µl of cDNA (diluted 1:30 in nuclease-free water), 10 µl of PerfeCTa SYBR® green Super Mix (Quanta Biosciences, Beverly, MA, USA), and 1 µl each of 10 µM forward and reverse primers in a 20 µl reaction volume. A Bio-Rad (Hercules, CA, USA) CFX Connect Real-Time System was used to run a temperature gradient of 95 °C for 3 min, then 36 cycles of 95 °C for 15 s, 58–68 °C for 30 s, with plate imaging after each cycle. A melt curve between 65 and 95 °C in 0.5 °C increments was obtained, with plate imaging after each increment to estimate PCR product number and primer specificity. Single products were observed for all primer pairs, with an optimum annealing temperature of 60 °C. Primer efficiency was tested using eight serial 2× dilutions of cDNA, with reaction conditions of 95 °C for 3 min, 36 cycles of 95 °C for 15 s, 60 °C for 30 s, and plate imaging after each cycle. Primer efficiency was between 1.93 and 2.13 (see Supplementary Table S3), and thus *C*_t_ values could be compared (Schmittgen and Livak, 2008). Amplification of cDNA from experimental samples was assessed with the same reaction conditions, using three 1994H cDNA samples, three 2015H cDNA samples, and no-reverse-transcriptase and no-template controls. Assays were performed in triplicate; results of three assays were averaged. *C*_t_ was calculated with Bio-Rad CFX Manager 3.1. Transcript integrity was calculated as Δ*C*_t_ (*C*_t_ of 3′-amplicon−*C*_t_ of 5′-amplicon). Average Δ*C*_t_ and standard deviation were calculated from biological replicates. A negative Δ*C*_t_ indicates that the 5′ end of the transcript is less abundant than the 3′ end, and thus the sample is degraded.

### Statistical analysis

All analyses were performed in Microsoft Excel (version 14) or JMP 12 (SAS Institute Inc., Cary, NC, USA). Variation around average values is expressed as ±standard deviation.

## Results

### Seed germination and RNA quality

**Fig. 1.**
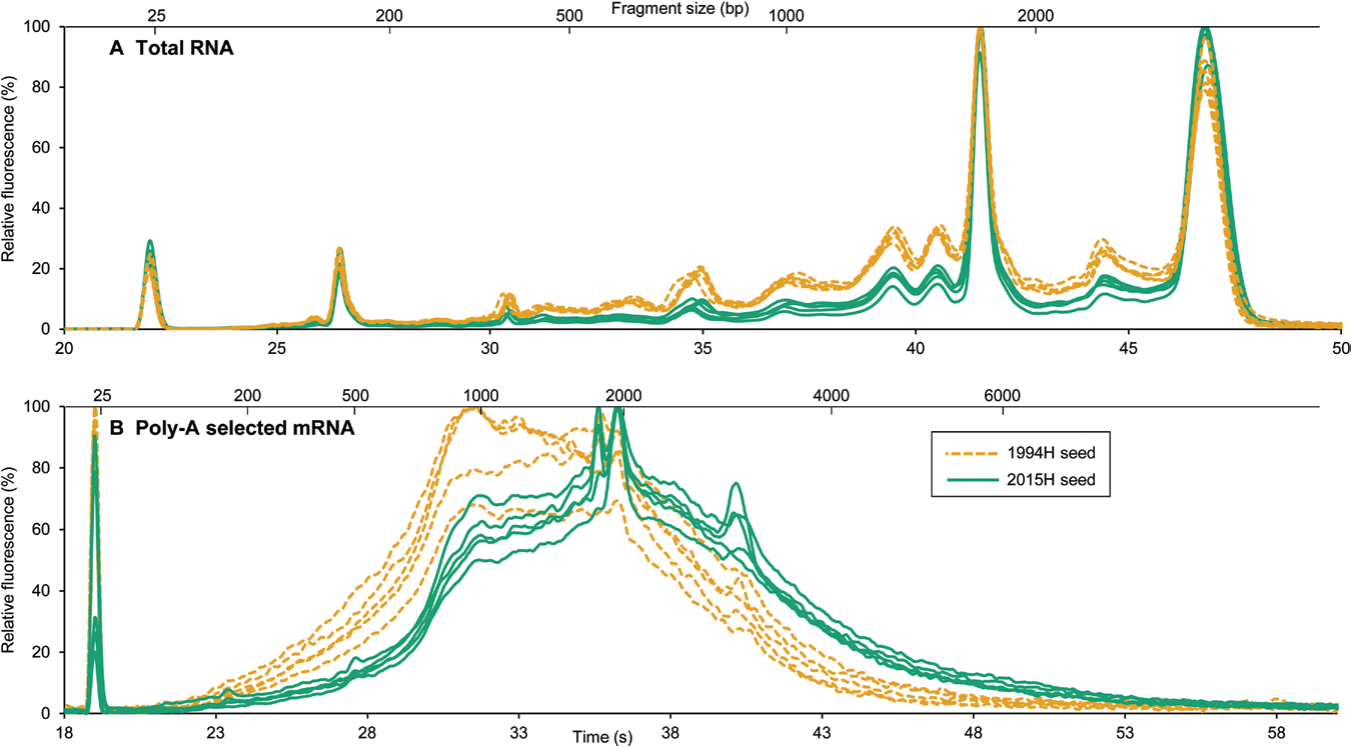
Electropherograms from five embryos of soybean seeds harvested in 1994 (stored 23 years, orange dashed lines) or 2015 (stored 2 years, green solid lines). (A) Total RNA from each sample. The differences in peak heights in 1994H relative to 2015H samples indicate RNA degradation. (B) DNAse-treated, poly-A RNA. The most abundant mRNAs are smaller in 1994H seeds than 2015H seeds.

Soybean seeds harvested in 2015 (2015H, stored 2 years) retained high germination (100% in 2016 and 2017), while 1994 harvested seeds (1994H, stored 23 years) showed signs of aging (61% germination in 2016, 0% in 2017) (Table 1). There was lower yield of RNA from 1994H (11.31 ± 1.04 µg mg^−1^ tissue) compared with 2015H embryonic axes (15.29 ± 2.49 µg mg^−1^ tissue) (*P*<0.05, Student’s *t*-test). Compared with 2015H seeds, electropherograms of total RNA from 1994H embryonic axes had a lower ratio of 25S and 18S rRNA peak heights (47 and 41.5 s, respectively), and greater heights of similarly sized peaks in the inter-peak (42.8–45.9 s) and fast (27.25–41 s) regions (Fig. 1A). The average RIN values (Schroeder *et al.*, 2006) for 1994H and 2015H samples were 6.72 ± 0.08 and 7.86 ± 0.34, respectively (*P*<0.0001, Student’s *t*-test) (Table 1). Yield of poly(A)-selected mRNA appeared less for 1994H compared with 2015H tissues, but the difference was not significant (*P*=0.16, Student’s *t*-test) (Table 1). Length of most of the poly(A)-selected mRNA from 1994H seed tissue was 1000–2000 bp (30–37 s), compared with 2015H seed tissue, which was 1000–3000 bp (30–41s) (Fig. 1B), indicating a shift towards lower molecular mass molecules.

**Table 1.** Germination percentage and RNA quality of seed lots used for RNA sequencing

Harvest year	Germination (%)		RNA yield (µg RNA mg^−1^ tissue)	RIN	mRNA yield (ng poly(A)-selected RNA mg^−1^ tissue)
	2016	2017			
2015	100	100	15.29 ± 2.49	7.86 ± 0.34	22.62 ± 5.67
1994	61	0	11.31 ± 1.04^*^	6.72 ± 0.08^**^	15.94 ± 7.68

Germination was measured in 2016 and 2017. RNA data show the mean ±SD (*n*=5) for RNA extracted from individual embryonic axes. *P<0.05, **P<0.0001 (Student’s *t*-test).

### Transcriptome curation for well-sequenced transcripts

Each MinION R9.4 flow cell sequenced over 2.1 million cDNA molecules (4908733 total events) that were synthesized from poly(A)-selected mRNA from five individual axes excised from 1994H and 2015H seeds. About 97% of molecules were converted to nucleotide sequences (reads), and 3559336 of those had unambiguous barcode sequences and were retained. There was no significant difference in the average number of reads between 1994H and 2015H samples (338599 ± 103013 and 373269 ± 120197, respectively) (*P*>0.3, Student’s *t*-test). In total, 3468518 (97%) reads mapped to one of 56137 transcripts in the *Glycine max_275_Wm82.a2.v1* reference transcriptome. A similar number of reads mapped to the reference transcriptome in all five 1994H or 2015H samples (1649345 and 1819173, respectively). Of 56137 reference transcripts, 34753 (62%) were found in at least one seed (see Supplementary Fig. S2A; Supplementary Dataset S1) and 27217 (48%) were found in at least one seed from both harvest years. There were 3409 transcripts found in at least one 1994H seed and no 2015H seeds, and 4127 transcripts found in at least one 2015H seed and no 1994H seeds. Eight transcripts were found in all five 1994H axes and no 2015H axes; conversely, nine transcripts were found in all five 2015H axes and no 1994H axes (Supplementary Table S4). We filtered transcripts to those found in all 10 axes, narrowing the transcript pool from 34753 to 11729 (Supplementary Fig. S2B). Of these, 23 were longer than 9000 bp (Supplementary Table S5), with poor coverage in all 10 samples. We then removed transcripts with inherent alignment problems by retaining transcripts with >75% coverage in 2015H seeds, reducing the transcript pool to 8787 (Supplementary Fig. S2C).

**Fig. 2.**
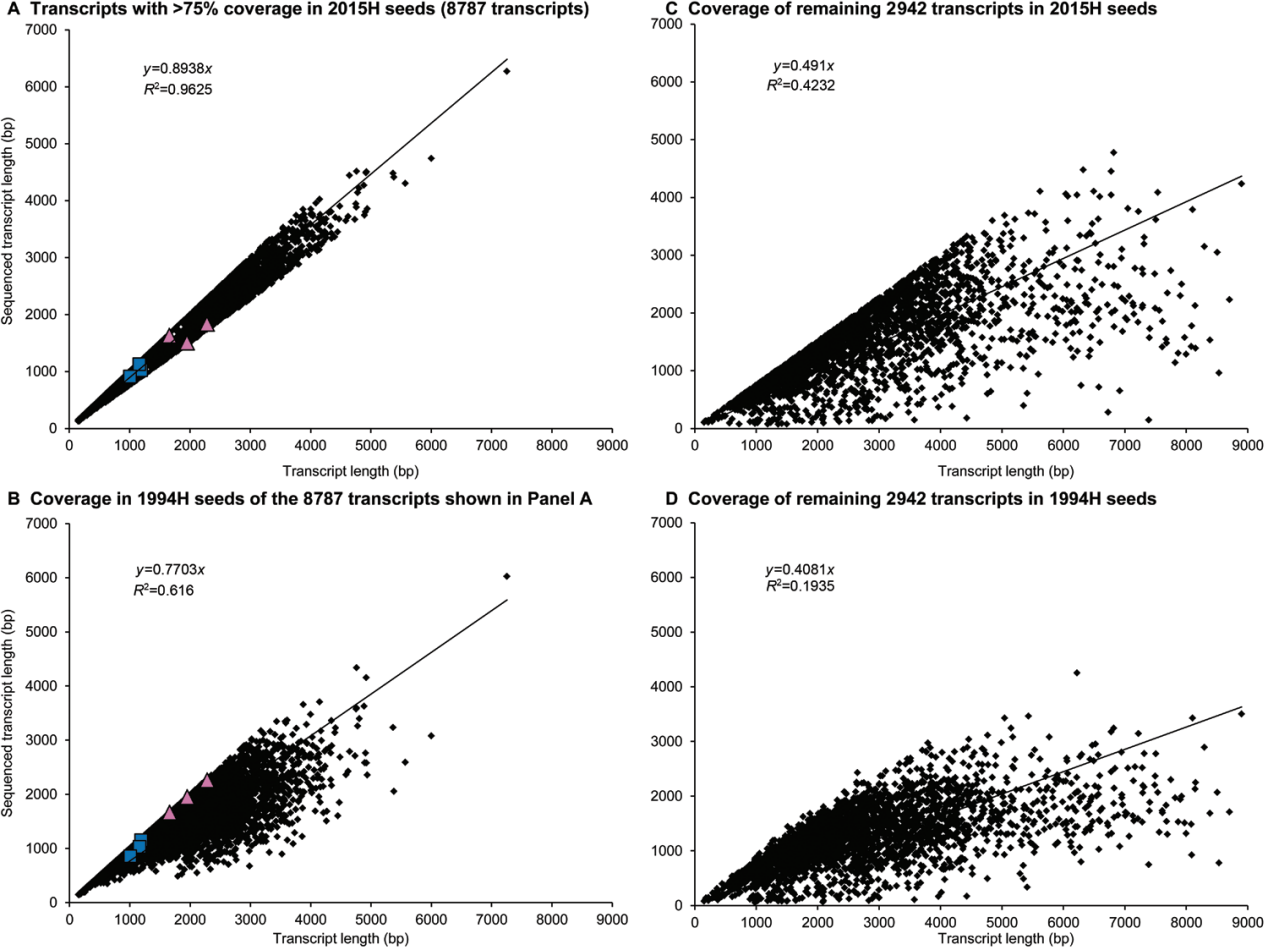
Comparison of reference and sequenced transcript lengths in the 11729 transcripts occurring in all 10 samples. (A) Sequenced transcript lengths in 2015H samples for transcripts where the read alignments from 2015H samples covered at least 75% of the reference transcript (8787 transcripts). (B) Sequenced transcript lengths for the same 8787 transcripts in 1949H samples. The six transcripts chosen for qPCR analysis are shown as blue squares (‘intact’ transcripts, Fig. 5 region A) or pink triangles (‘degradation-prone’ transcripts, Fig. 5 region B). (C, D) Sequenced transcript lengths for the remaining 2942 transcripts are shown in 2015H samples (C) and 1994H samples (D). A significant linear correlation between actual and sequenced transcript length exists in all cases, with the strongest correlation in (A).

Lengths of reference and sequenced transcripts were positively correlated [2015H: *R*^2^=0.96, *y*=0.89*x*, *P*<0.0001 (Fig. 2A); 1994H: *R*^2^=0.68, *y*=0.77*x*, *P*<0.0001 (Fig. 2B), *n*=8787]. The weaker correlation for 1994H indicates shorter transcript lengths than expected, particularly for longer transcripts (Fig. 2B). Reference and sequenced transcript length strongly correlated in both cohorts (*R*^2^>0.95, 6988 transcripts) when >75% coverage for 1994H samples was the filtering criterion (see Supplementary Fig. S3). Between 22 and 100% (median of 86%) of 1994H transcripts aligned when the filtering criterion was >75% coverage for 2015H samples. Conversely, about 93% of 2015H transcripts aligned when the filtering criterion was >75% coverage for 1994H samples. Hence, there was unequal representation of full transcript lengths between 1994H and 2015H seed tissues. Lengths of reference and sequenced transcripts were weakly correlated for the 2942 transcripts with <75% coverage (see lower *R*^2^ and slopes, Fig. 2C, D).

The subset containing 8787 transcripts was further filtered to contain only transcripts that were deeply sequenced, where at least one base had sequencing depth ≥20. These remaining 2211 transcripts (‘curated transcripts’, Supplementary Fig. S2D) were considered abundant and contained high-quality sequences, allowing statistical comparison between cohorts.

### Degradation patterns in mRNA

**Fig. 3.**
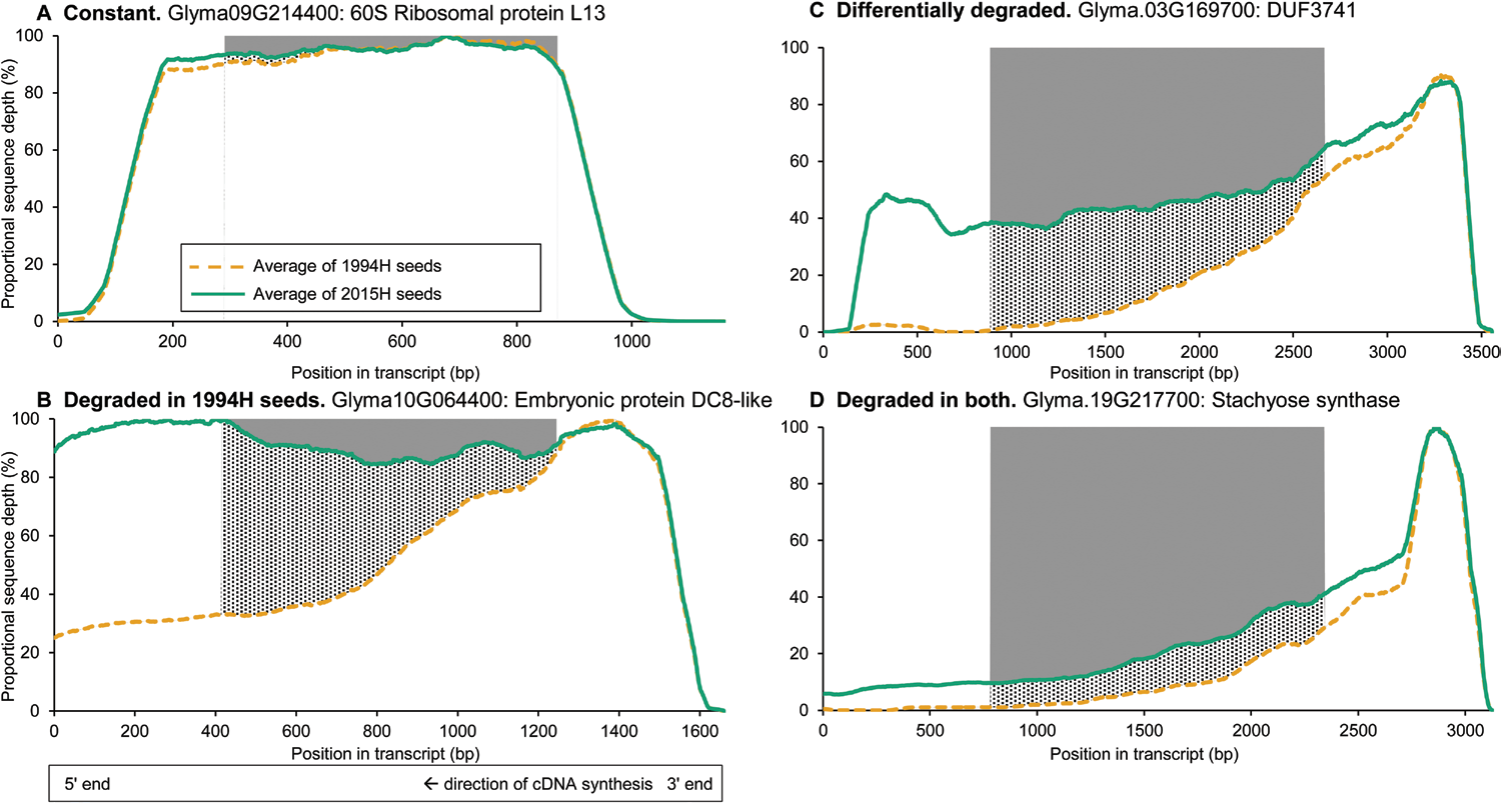
Comparison of average normalized sequence depth between reads from 2-year-old seeds (2015H, green solid line) and 23-year-old seeds (1994H, orange dashed line) at each position in a transcript. For this figure only, sequence depth was averaged across a moving window of 100 base pairs. (Examples of unsmoothed data appear in Supplementary Fig. S5.) Because of inaccurate alignments at transcript ends, only bases between 25 and 75% of the reference transcript length were considered. The shaded area indicates relative degradation (RD) of 2015H sequence compared with an intact transcript (RD_2015_), while the hatched area indicates RD of 1994H compared with 2015H sequence (RD_Δ_). Four common patterns were observed. (A) Constant coverage: observed sequence depth was approximately the same for each base in both cohorts. (B) Degradation in 1994H seeds relative to 2015H seeds: observed sequence depth is constant in 2015H seeds, but increases 5′ to 3′ in 1994H seeds. (C) Differential degradation between the two cohorts: observed sequence depth increases 5′ to 3′ in both cohorts, with a smaller increase in 2015H than 1994H seeds. (D) Degradation of coverage in reads from all samples: sequence depth increases 5′ to 3′ at approximately the same rate in both cohorts.

**Fig. 4.**
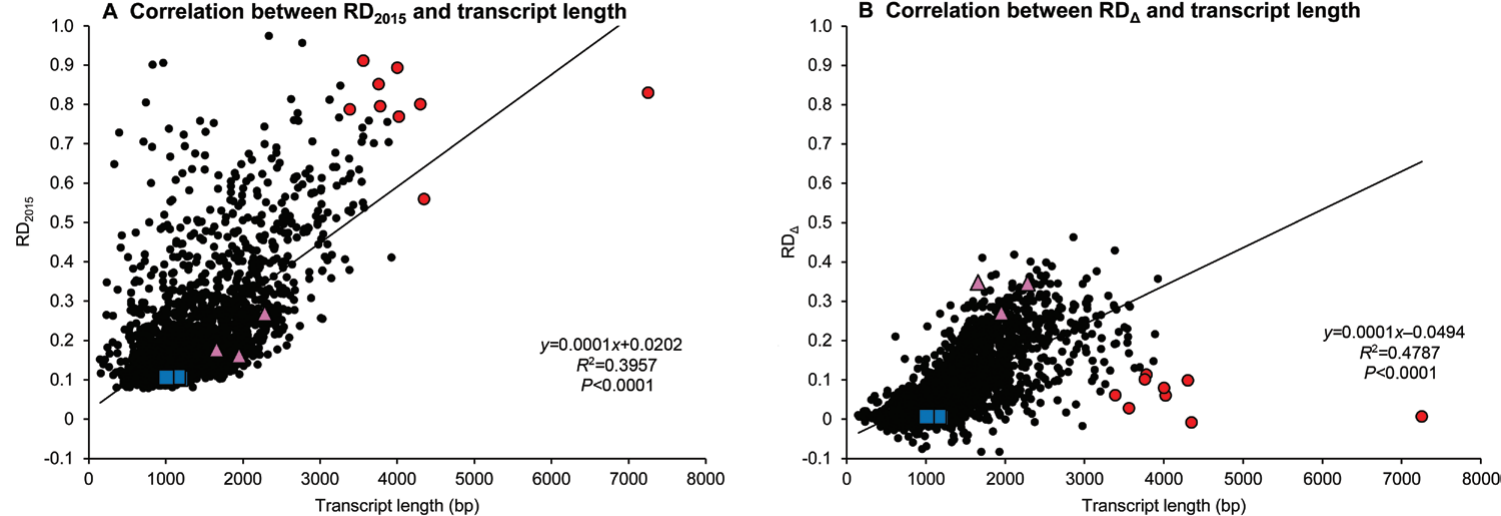
Relationship between transcript length, relative degradation (RD) of transcripts in 2015H samples compared with intact transcripts (RD_2015_), and transcripts in 1994H samples compared with 2015H samples (RD_Δ_). (A) The correlation between transcript length and RD_2015_ is significant but poorly predictive. (B) The correlation between transcript length and RD_Δ_ is significant and more strongly predictive. Transcripts chosen for qPCR analysis are shown as blue squares (‘intact’ transcripts, Fig. 5 region A) and pink triangles (‘degradation-prone’ transcripts, Fig. 5 region B). Nine transcripts with smaller RD_Δ_ than expected from transcript length are shown in red in both panels.

Degradation patterns were categorized among the 2211 curated transcripts by examining the number of reads at each base (Fig. 3). Intact transcripts showing no evidence of degradation exhibited near-constant read depth in both 2015H and 1994H samples [Fig. 3A, RD of 1994H compared with 2015H (RD_Δ_)≈0; RD of 2015H compared with a perfectly intact transcript (RD_2015_)≈0]. Transcripts that degraded during storage had constant read depth in 2015H samples and increasing read depth towards the 3′ end in 1994H samples (Fig. 3B, RD_Δ_>0, RD_2015_≈0), or increasing read depth of both cohorts, but at different rates (Fig. 3C, RD_Δ_>0, RD_2015_>0). Transcript length correlated with degradation, regardless of storage time (Fig. 4).

Similar rates of increasing read depths in 2015H and 1994H samples signaled transcript degradation unrelated to storage (Fig. 3D, RDΔ≈0, RD2015>0). These appeared as higher degradation in short transcripts (<3000 bp) from 2015H samples than would be expected by length alone (larger RD_2015_) (Fig. 4A, points above regression line), or similar degradation in long transcripts (>3000 bp) in both 2015H and 1994H samples (high RD_2015_ and low RD_Δ_, Fig. 4B). The longest transcript, Glyma.14G078300 (7255 bp, no annotation), was degraded in both cohorts (high RD_2015_, Fig. 4A).

**Fig. 5.**
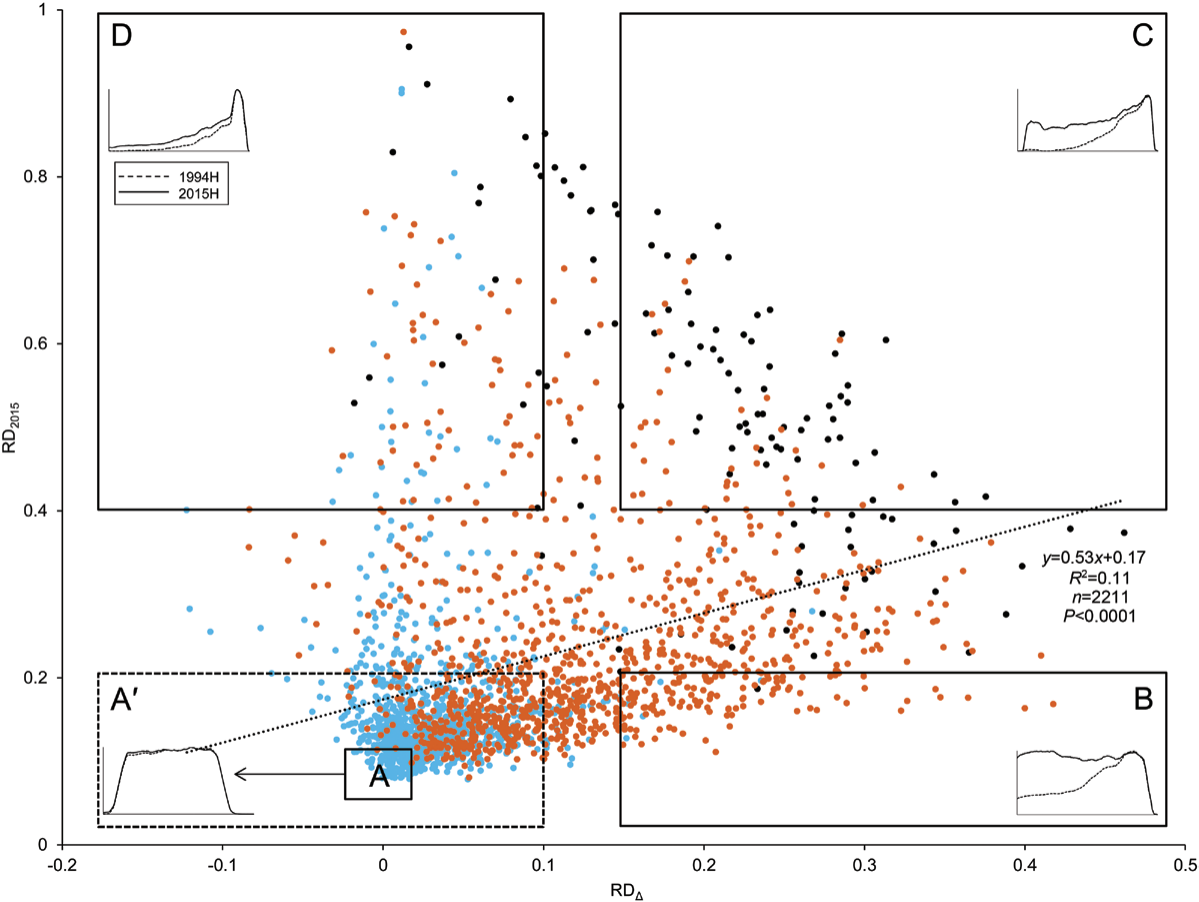
Relationship between degradation that occurs with time (relative degradation (RD) of 1994H samples compared with 2015H samples, RD_Δ_) and degradation that occurs early in storage (RD of 2015H samples compared with a perfectly intact transcript, RD_2015_). The four different patterns of transcript coverage described in Fig. 3 occur in different regions of this figure, and the associated pattern is indicated by a cartoon, where the proportional sequence depth is shown by a solid line for 2015H samples and a dashed line for 1994H samples. Regions were defined by the following boundaries: region A follows the minimum and maximum RD_Δ_ and RD_2015_ values for the ERCC Spike-In transcripts (−0.028<RD_Δ_<0.02; 0.06<RD_2015_<0.12) and encloses 170 transcripts; region A′, RD_Δ_<0.1 and RD_2015_<0.2, 1149 transcripts; region B, RD_Δ_>0.15, RD_2015_<0.2, 114 transcripts; region C, RD_Δ_>0.15, RD_2015_>0.4, 99 transcripts; region D, RD_Δ_<0.1 and RD_2015_>0.4, 104 transcripts. Transcript length is indicated by color as follows: black, transcript >2500 bp; orange, 1200 bp<transcript<2500; blue, transcript<1200 bp. Stratification by transcript size is evident, as most transcripts <1200 bp have small RD_Δ_ and RD_2015_, most transcripts >2500 bp have large RD_Δ_ and RD_2015_, and remaining transcripts are intermediate.

Comparisons between transcript coverage for 2015H (RD_2015_) and 1994H (RD_Δ_) samples indicate transcripts that degraded early on (RD_2015_>>0) and with increasing storage time (RD_Δ_>>0) (Fig. 5). Shorter transcripts (<1200 bp, Fig. 5, blue circles) were more stable (both RD_2015_ and RD_Δ_≈0) than longer transcripts (>2500 bp, Fig. 5, black circles) (RD_2015_>0.4 and RD_Δ_>0.15), suggesting continuous degradation. The significant but weak correlation between RD_2015_ and RD_Δ_ (*R*^2^=0.11; *P*<0.0001) suggests different patterns for aging-related versus aging-unrelated mRNA degradation, which are categorized in Fig. 3 and portrayed in different quadrants of Fig. 5. Region A (−0.028<RD_Δ_<0.02 and 0.06<RD_2015_<0.12; boundaries defined by intact ERCC Spike-In control transcripts) encompasses 175 transcripts with no signs of deterioration after 23 years of storage. Most (172) of these transcripts are <1200 bp; all are <1300 bp (see Supplementary Table S6). A larger region A′ (RD_Δ_<0.1 and RD_2015_<0.2) encompasses 1149 transcripts, or 52% of the data set. Most (853) are <1200 bp; the remaining 296 have intermediate lengths (1200–2500 bp, Fig. 5, orange circles). Region B (RD_Δ_>0.15 and RD_2015_<0.2) encompasses 114 transcripts that appear intact in 2015H samples and degraded in 1994H samples. Most (108) have intermediate length. Five short transcripts (737–1112 bp) and one long transcript (2572 bp) show degradation patterns uncharacteristic of their size classes (Supplementary Table S6). Region C (RD_Δ_>0.15 and RD_2015_>0.4) encompasses 99 transcripts showing some degradation in 2015H samples and more degradation in 1994H samples. Region C transcripts were long (61) or intermediate (38). These long transcripts represent almost half of the 126 transcripts >2500 bp in the dataset. None of the 1042 transcripts <1200 bp was located in region C. Finally, region D (RD_Δ_<0.1 and RD_2015_>0.4) encompasses 104 transcripts that appear to degrade initially, with little further change during 23 years of storage. Most (64) of these transcripts exhibited alternative splicing-like patterns (Abdel-Ghany *et al.*, 2016), with inflated RD_2015_ values due to missing exons, and low RD_2015_ and RD_Δ_ values for present exons (Supplementary Fig. S4). Region D includes transcripts of all sizes, ranging from 335 to 7255 bp (33 transcripts <1200 bp, 17 transcripts >2500 bp).

We investigated alternatives to transcript length to explain the distribution of transcripts in Fig. 5. Average GC content, which is associated with transcript stability (Gallego Romero *et al.*, 2014; Zhao *et al.*, 2017), was slightly, but significantly, different among the four regions, ranging between 42% in region D and 44% in region B (see Supplementary Table S7). Comparisons of frequency of GO terms between our dataset (34753 transcripts, 27755 annotated) and another using soybean embryonic axes (36929 transcripts, 31874 annotated) (Bellieny-Rabelo *et al.*, 2016) showed no significant differences in any of the three ontological domains (molecular function, biological process, cellular component), indicating that our transcriptome was representative of the embryonic axis transcriptome. We also applied the over-representation test among transcripts found in each of the four regions of Fig. 5. About half of the 1878 transcripts were annotated as ‘unclassified’ (Table 2). Among transcripts in region A (no degradation), twenty GO terms were significantly over-represented, related primarily to ribosomal function and translation (Table 2; Supplementary Dataset S2). Region C transcripts (slight degradation in 2015H seed tissue, pronounced degradation in 1994H seed tissue) had significant over-representation of ATP binding terms and related functions (Supplementary Table S8).

**Table 2.** Gene ontology terms over-represented in transcripts found in region A (low RD_2015_ and RD_Δ_; ‘intact’ transcripts), identified by the PANTHER over-representation test using a Bonferroni correction for multiple testing

GO	**Transcript annotation**	**GO ID**	**Ref.**	**Reg. A**	**FE**	***P***
**MF**	**Structural constituent of ribosome**	**GO:0003735**	**215**	**32**	**2.17**	**0.006**
MF	Structural molecule activity	GO:0005198	219	32	2.13	0.008
MF	Unclassified		705	52		
**BP**	**Translation**	**GO:0006412**	**265**	**36**	**1.98**	**0.01**
BP	Cellular macromolecule bp	GO:0034645	320	42	1.91	0.005
BP	Macromolecule bp	GO:0009059	320	42	1.91	0.005
BP	Peptide bp	GO:0043043	267	36	1.96	0.02
BP	Peptide mp	GO:0006518	274	37	1.97	0.01
BP	Cellular amide mp	GO:0043603	278	37	1.94	0.02
BP	Amide bp	GO:0043604	268	36	1.96	0.02
BP	Cellular nitrogen compound bp	GO:0044271	334	41	1.79	0.03
BP	Gene expression	GO:0010467	387	46	1.73	0.02
BP	Unclassified		794	45		
**CC**	**Cytosolic ribosome**	**GO:0022626**	**180**	**30**	**2.43**	**0.0007**
CC	Ribosome	GO:0005840	224	33	2.15	0.003
CC	Intracellular ribonucleoprotein complex	GO:0030529	309	38	1.79	0.03
CC	Ribonucleoprotein complex	GO:1990904	309	38	1.79	0.03
CC	Intracellular non-membrane-bounded organelle	GO:0043232	298	40	1.96	0.002
CC	Non-membrane-bounded organelle	GO:0043228	298	40	1.96	0.002
CC	Cytosolic part	GO:0044445	186	30	2.35	0.001
CC	Cytosol	GO:0005829	228	34	2.17	0.001
**CC**	**Ribosomal subunit**	**GO:0044391**	**194**	**30**	**2.25**	**0.003**
CC	Unclassified		829	47		

Annotations are ordered hierarchically, with the most specific (child) annotations listed in bold. BP, biological process; bp, biosynthetic process; CC, cellular component; FE, fold enrichment; MF, molecular function; mp, metabolic process; Ref., number of the 1821 annotated curated transcripts with this annotation; Reg. A, number of the 125 annotated transcripts in region A with this annotation.

### Oxidation of guanosine

**Fig. 6.**
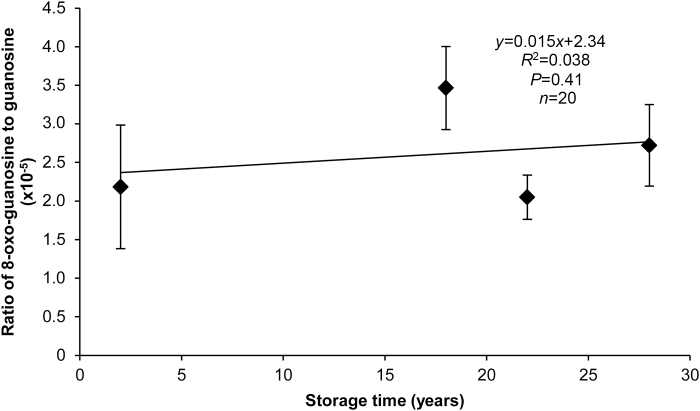
RNA oxidation in soybean embryonic axes stored for 2–28 years, expressed as the ratio of 8-oxo-guanosine to guanosine. Each point represents the average of values from five seeds; error bars show standard deviation. There is no significant linear correlation between storage time and relative abundance of 8-oxo-guanosine.

Transcript degradation from the 5′ end could reflect age-related RNA oxidation that interfered with reverse transcription (Gong *et al.*, 2006). The ratio of abundance of the most easily oxidized base, 8-oxo-guanosine (Kong and Lin, 2010) to abundance of guanosine in total RNA did not correlate with storage time for embryonic axes of soybean seeds harvested in 1989, 1995, 1999, and 2015 (Supplementary Table S9) (*y*=0.013*x*+2.38, *R*^2^=0.028, *P*=0.48, *n*=20) (Fig. 6).

### qPCR confirmation of fragmentation

**Fig. 7.**
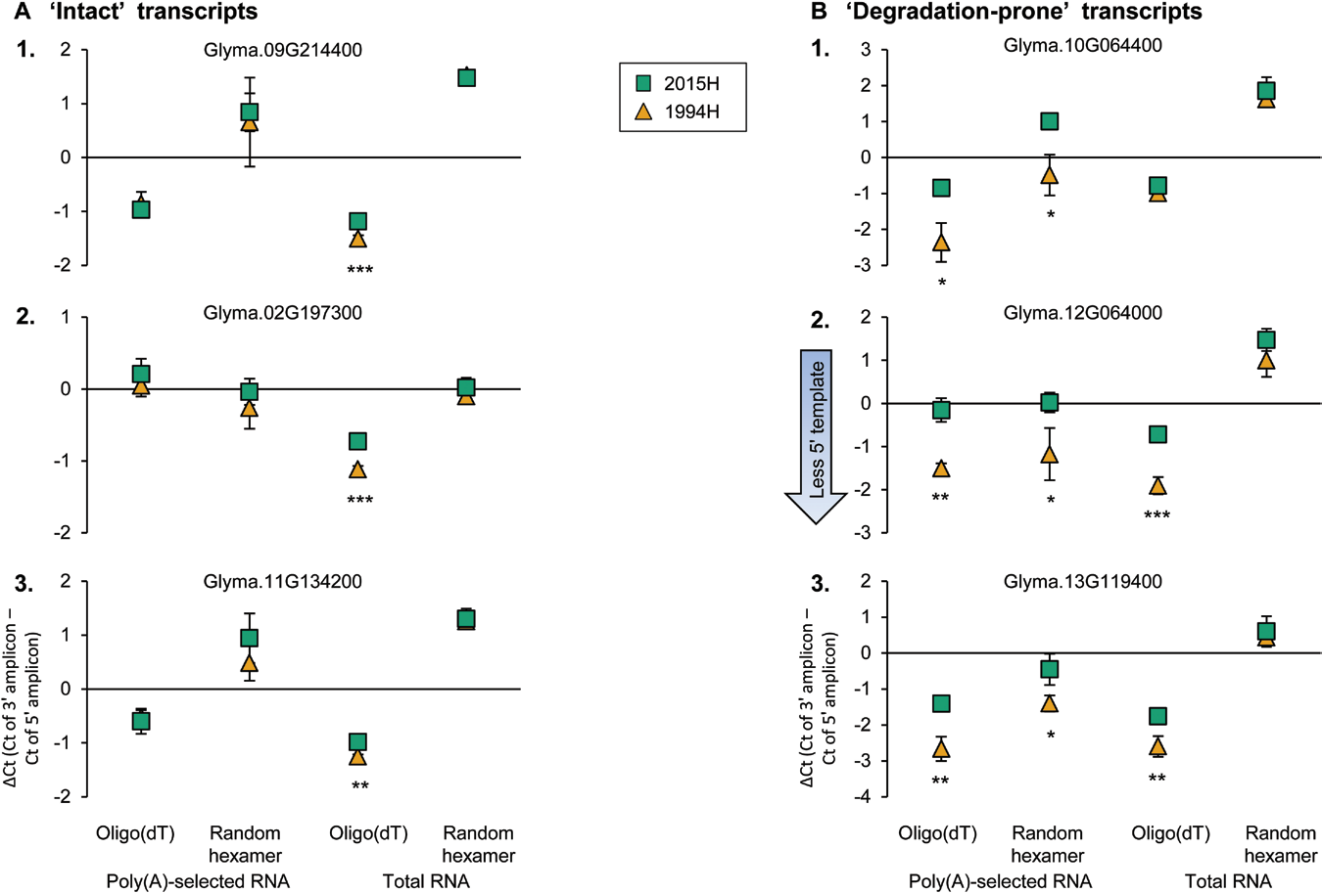
Confirmation of transcript degradation using qPCR. Δ*C*_t_ values were calculated between the *C*_t_ values of 3′ and 5′ amplicons from the average of three replicate assays performed on three seeds from the same harvest year, ±standard deviation; green squares, 2015H; orange triangles, 1994H. A negative Δ*C*_t_ means the 5′ amplicon template is less abundant than the 3′ amplicon template in the cDNA library. Four cDNA libraries were tested, using oligo(dT) and random hexamer primers and poly(A)-selected and total RNA. (A) Three ‘intact’ transcripts (Fig. 5, region A) were tested: 1, Glyma.09G214400; 2, Glyma.02G197300; 3, Glyma.11G134200. In each library, both cohorts show similar Δ*C*_t_ values for each transcript. (B) Three ‘degradation-prone’ transcripts (Fig. 5, region B) were tested: 1, Glyma.10G064400; 2, Glyma.12G064000; 3, Glyma.13G119400. The two cohorts have significantly different Δ*C*_t_ values in the three libraries using poly(A) selection or oligo(dT) priming, but have similar Δ*C*_t_ values for the library made from random hexamer-primed total RNA. Significance (Student’s *t*-test) is indicated by asterisks: *P<0.05, **P<0.01, ***P<0.001.

To confirm mRNA fragmentation, quantitative PCR was used to compare the integrity of three transcripts from region A (no degradation) and three from region B (pronounced degradation only in 1994H seed tissue). Integrity was measured as relative amplification at the 5′ and 3′ ends (Δ*C*_t_=*C*_t_ of 3′ amplicon−*C*_t_ of 5′ amplicon) in poly(A)-selected or total RNA cDNA libraries synthesized using oligo(dT) or random hexamer reverse-transcription primers (Fig. 7; Supplementary Figs S5, S6, S7; Supplementary Tables S7, S8). For region A transcripts, amounts of template cDNA for 5′ and 3′ amplicons were similar among cohorts and cDNA libraries (Fig. 7A). The three cDNA libraries synthesized using random hexamer primers and/or poly(A) RNA as starting material showed no differences in Δ*C*_t_ values (*P*>0.2 for all three transcripts in each library, Student’s *t*-test). For the oligo(dT)-primed total RNA cDNA library, significant but small differences between 2015H and 1994H samples were observed (Δ*C*_t_ values of −1.18 ± 0.01, −0.72 ± 0.05, and −0.98 ± 0.05 in 2015H samples; −1.50 ± 0.06, −1.11 ± 0.04, and −1.25 ± 0.04 in 1994H samples for transcripts 1, 2, and 3, respectively; *P*<0.002 for all transcripts, Student’s *t*-test).

In contrast to intact transcripts, Δ*C*_t_ values differed significantly between cohorts for degradation-prone transcripts (region B) in libraries made from poly(A)-selected RNA. In 1994H samples, 5′ amplicon template was less abundant than 3′ amplicon template, generating negative Δ*C*_t_ values. In 2015H samples, templates for both amplicons were similarly abundant (*P*<0.04, Student’s *t*-test, Fig. 7B). A greater difference in Δ*C*_t_ values in 1994H samples was also seen in the oligo(dT)-primed total RNA cDNA library [transcript 1 Δ*C*_t_=−0.79 ± 0.06 in 2015H and −0.99 ± 0.14 in 1994H, *P*=0.08 (not significant); transcript 2 Δ*C*_t_=−0.72 ± 0.04 in 2015H and −1.91 ± 0.2 in 1994H, *P*=0.0005; transcript 3 Δ*C*_t_=−0.45 ± 0.44 in 2015H and −1.41 ± 0.23 in 1994H, *P*=0.01, Student’s *t*-test]. However, for the random hexamer-primed total RNA cDNA library, there were no observed differences between cohorts; Δ*C*_t_ values were all positive (Δ*C*_t_ values in 2015H samples of 1.85 ± 0.38, 1.47 ± 0.26, and 0.60 ± 0.42 and in 1994H samples of 1.62 ± 0.15, 1.00 ± 0.38, and 0.43 ± 0.22 for transcripts 1, 2, and 3, respectively; *P*>0.1 for all three transcripts, Student’s *t*-test).

## Discussion

Degradation of mRNA was compared in embryonic axes of seeds harvested 21 years apart that showed no (2015H) and advanced (1994H) symptoms of aging. RNA was sequenced using whole-molecule cDNA sequencing without fragmentation. We found that some transcripts were degraded in both cohorts, but substantially more damage was observed in tissues from 1994H seeds. Damage occurred mostly by fragmentation, as seen in changes in electropherogram profiles (Fig. 1), length of transcript coverage (Fig. 2), and depth of sequencing (Figs 3–5). There was little evidence that transcripts were lost (Supplementary Tables S4, S5) or incurred oxidative lesions (Figs 6, 7B). The continuous distribution of fragment sizes in degraded transcripts and positive correlation between transcript length and degradation support the hypothesis that mRNA degradation occurs by random fragmentation, which is consistent with hypotheses that mortality arises from oxidation events (Hendry, 1993; Burrows and Muller, 1998; Kranner, 2005).

### RNA damage likely occurs through non-enzymatic fragmentation

RNA damage via non-enzymatic oxidation of bases is associated with aging in humans (Honda *et al.*, 2005) and dormancy breaking during dry after-ripening in seeds (Bazin *et al.*, 2011; Gao *et al.*, 2013). We saw no evidence of base oxidation (Fig. 6), and this is supported by our qPCR studies showing no blockage of oligo(dT)-primed cDNA synthesis (Gong *et al.*, 2006). Instead, degraded transcripts were present in their entirety, but as fragments, reflected in similar Δ*C*_t_ values for both cohorts in the cDNA library made from random hexamer-primed total RNA (Fig. 7B). It is unlikely enzymatic digestion of the poly(A) tail, which normally initiates turnover, occurred during dry storage, because polyadenylated mRNA selection yielded similar numbers of transcripts in tissues of both 1994H and 2015H seeds (30626 and 31344, respectively). Transcript length was strongly and positively correlated with percent coverage (Figs 2, 4, 5) which continuously increased from the 5′ to 3′ end of the transcript (Fig. 3), suggesting that breaks occurred at random positions. The non-specific pattern of molecule degradation suggests that enzymatic catalysis was not a factor (Garneau *et al.*, 2007). Collectively, the pattern of degradation, indication of non-enzymatic degradation signaling a metabolic switch, and low probability of substrate diffusion to enzyme active sites in solid-state systems (Walters, 1998, 2005; Walters *et al.*, 2010, and references therein) strongly argue that the RNA damage we observed occurred by random oxidative attack to the molecule rather than enzymatic catalysis.

### Four patterns of degradation

We recognized four patterns of transcript degradation based on sequence coverage and timing of degradation. Many of the shortest (<1200 bp) transcripts were relatively intact (regions A and A′, Fig. 5) after 23 years. Transcripts having ribosomal functions, particularly translation, were over-represented in this group (Table 2) and may indicate the importance of reconstituting translational machinery during germination (Rajjou *et al.*, 2008). However, high integrity of these transcripts did not prevent 1994H seeds from dying, suggesting these gene functions are not sufficient to ensure seed viability. Transcripts that were intact in fresh seeds and degraded in 1994H seeds (region B, Fig. 5) have a higher potential to mark the progress of aging and eventual loss of viability. Most of these transcripts had lengths between 1200 and 2500 bp and represented a range of functions, likely reflecting the functional diversity required for germination. Comparisons among cohorts and species may reveal differences in degradation rates of shorter and longer transcripts, providing an insight into longevity differences. Transcripts that were somewhat degraded in 2015H seeds and more degraded in 1994H seeds (region C, Fig. 5) are usually long (>2500 bp). These transcripts are candidate markers for aging before seeds lose viability. Finally, there was also a group of transcripts from all length classes that degraded to the same extent in both cohorts (region D, Fig. 5), indicating fragmentation occurred soon after maturation drying. These transcripts might encode proteins that are not involved in germination, or that were already translated and available to the seed at the onset of imbibition. Their degradation may result from lack of protection during maturation drying, or from targeted, but incomplete, degradation at maturity. The strong degradation observed in fresh seeds resembles ‘long-lived decay intermediates’ in wild oat that are retained through drying and after-ripening (Johnson *et al.*, 1999; Johnson and Dyer, 2000). As with the transcripts categorized here, ‘decay intermediates’ in oat showed a continuous size distribution across the entire transcript length (e.g. Fig. 3D).

### Longevity-associated transcripts

Our work differs from several current studies seeking genes associated with longevity (Bentsink *et al.*, 2010; Schwember and Bradford, 2010; Pereira Lima *et al.*, 2017). The persistence of longevity-associated transcripts might not be necessary in dry seeds when the gene product is already present and required for cell survival during quiescence (Berjak *et al.*, 1989; Hay *et al.*, 2010; Pereira Lima *et al.*, 2017). Longevity products likely include antioxidants (Sattler *et al.*, 2004; Kranner, 2005), protein repair enzymes (Ogé *et al.*, 2008; Petla *et al.*, 2016), heat shock proteins (Dell’Aquila *et al.*, 1998; Pereira Lima *et al.*, 2017), and proteins involved in chloroplast functions. Of the 27 longevity-associated transcription factors identified in maturing soybean seeds (Pereira Lima *et al.*, 2017), 25 occurred in our dataset from dry soybean (Supplementary Dataset S1), and seven were retained in the curated transcripts. These seven transcripts (1180–3372 bp) were relatively intact in 2015H seeds (low RD_2015_, Fig. 5), but exhibited broad variation in integrity in 1994H seeds, from minor damage to nearly complete degradation. We could not discern a degradation pattern for the 1525 genes that were down-regulated in association with increased longevity (Pereira Lima *et al.*, 2017). However, in fresh seeds, the 742 genes that were up-regulated in association with increased longevity were relatively intact (low RD_2015_). These also showed a range of integrity in the 1994H cohort, and so were mostly categorized in region B (Fig. 5). One transcript (Glyma.03G159000) was degraded in both cohorts (region D, Fig. 5), and encodes an α/β-hydrolase superfamily protein. Hence, our work identifies some longevity-associated transcripts that appear to degrade contemporaneously with loss of viability. This subset of transcripts provides an interesting opportunity to identify proteins essential for resumption of healthy metabolism and germination.

Stored mRNA is used for germination in healthy seeds (Bai *et al.*, 2017) and is sufficient for germination when transcription is blocked (Rajjou *et al.*, 2004; Sano *et al.*, 2012). We do not know whether fragmented mRNA impedes resumption of metabolism when the dry, quiescent organism rehydrates. Fragmented transcripts are likely to slow translation and generate dysfunctional proteins, if they are translated at all (Graille and Séraphin, 2012), and translation is required for germination (Rajjou *et al.*, 2004; Sano *et al.*, 2012). Damage to translational proteins and total RNA is associated with loss of germination ability during storage (Holdsworth *et al.*, 2008; Rajjou *et al.*, 2008, Fleming *et al.*, 2017) and mRNA fragmentation may be another component of the translational machinery related to longevity. Inability to recover degraded transcripts essential for germination may cause mortality.

### Prospects for understanding aging mechanisms and identifying aging markers

Long-lived seeds are quiescent and retain the machinery needed for germination through either protection or subsequent repair (Wood and Oliver, 1999; Ogé *et al.*, 2008; Kushwaha *et al.*, 2013; Waterworth *et al.*, 2015; Petla *et al.*, 2016). The process of aging undermines critical cell functions during quiescence. We initiated the study to identify molecules that mark the progress of deterioration by degrading while seeds lose viability, and to potentially link these to critical gene products or cell functions during germination. The granularity of sequence data presented here provides optimism for this objective through future discovery of degradation fates for key transcripts.

All unprotected molecules are targets for oxidative activity in quiescent cells, and so it is logical to propose a signature of aging in which the earliest signs are reflected by degradation products from the largest or most prevalent molecules. Conversely, a long and intact transcript would provide evidence of protection. Further tests of both the signature and signs of protection are possible through transcript-level analysis of seeds from our legacy collection that contains seventeen cohorts of ‘Williams 82’ soybean (Fleming *et al.*, 2017), and similar age classes for 40 other species.

Seeds eventually die during storage, and the rate is regulated by moisture and temperature of the storage environment. The rate might also be manifested by the frequency of random events such as degradation of mRNA, as we report here. Degradation of random molecules at random loci is an idea congruent with classical concepts of aging. Nevertheless, seeds in an apparently uniform population die at different rates. This non-linear kinetic can be explained by chance damage or escape from damage of molecules critical to germination. Hence, there should be an imperfect but positive correlation between random damage measured by RIN or transcript coverage, and seed mortality. The general pattern of degradation at random sites provides a powerful tool to measure aging rate before physiological symptoms are apparent. The element of chance also provides the potential to identify differential degradation of transcripts that lead to the different germination capacities among aging seeds.

## Supplementary data

Supplementary data are available at *JXB* online.

Methods S1. Detailed description of Oxford Nanopore cDNA synthesis and sequencing library preparation.

Dataset S1. Transcripts identified in each embryonic axis.

Dataset S2. Transcripts annotated with over-represented GO terms in Fig. 5, region A.

Fig. S1. Quality control of sequence data based on ERCC transcripts.

Fig. S2. Summary of transcript filtering process.

Fig. S3. Comparison of sequenced transcript length to actual transcript length in the 11729 transcripts sequenced in all 10 samples.

Fig. S4. Example of alternative splicing-like coverage pattern.

Fig. S5. Coverage maps of transcripts chosen for qPCR assay.

Fig. S6. Schematic representation of transcripts and amplicons.

Fig. S7. Expected qPCR results.

Table S1. Genes and amplicons chosen for qPCR.

Table S2. Primer sequences for each qPCR amplicon.

Table S3. *C*_t_ values and efficiency calculations for each qPCR primer set.

Table S4. Transcript names and annotations for transcripts exclusive to 1994H or 2015H seeds.

Table S5. Transcripts longer than 9000 bp sequenced in all 10 samples.

Table S6. Transcripts with unexpected decay profiles given their length.

Table S7. GC content in each region of Fig. 5.

Table S8. Over-represented GO terms for region C in the molecular function GO domain.

Table S9. Final germination percentage and RNA quality of seed lots used for RNA oxidation measurements.

Supplementary Figures Tables

ery215_suppl_Supplementary_Data_Sets

## Abbreviations:

GO: Gene Ontology
RD: relative degradation
RIN: RNA integrity number
