# Supplementary material for: Exploring the fate of mRNA in aging seeds: protection, destruction, or slow decay?
Source: J Exp Bot. 2018 Jun 12;69(18):4309–21. doi: 10.1093/jxb/ery215 (PMC6093385; doi:10.1093/jxb/ery215)
Supplement: Supplementary Figures Tables [file jxb_69_18_4309_s8.pdf]

Supplementary Figure Legends

Figure S1. Quality control of sequence data based on ERCC transcripts. A) Average number of counts across all ten samples plotted for each transcript abundance class (22 classes, ranging from 0.0002 to 600 amol µL-1 in the dilution used for sequencing). The relationship is strongly linear, indicating that more abundant transcripts are more deeply sequenced, both for ERCC transcripts and soybean transcripts. Error bars show standard deviation. B) Variance in average number of counts across all ten samples for transcript ID (diamonds) or abundance class (squares). The data are strongly related by a quadratic function, indicating that error in the number of counts is more related to the abundance of that transcript than to any variance among the ten samples.

Figure S2. Summary of transcript filtering process. Each panel shows the number of soybean transcripts retained after each step. Embryonic axis color indicates harvest year: 1994 (orange) and 2015 (green). Numbers in parentheses indicate how many transcripts were retained from the 92 transcripts in the ERCC Spike-In mix, which have different sequence lengths (from 273 to 2,022 bp), GC contents (from 30.8 to 52.7%), and abundances (from 0.0003 to 600 amol μL^{-1}, as diluted for sequencing).

Figure S3. Comparison of sequenced transcript length to reference transcript length in the 11,729 transcripts sequenced in all 10 samples. A) Transcripts where the read alignments from 1994H samples covered at least 75% of the reference transcript (6,988 transcripts). B) Sequenced transcript length of the same 6,988 transcripts in 2015H samples. Strong positive linear correlations hold in both cases, with slope ≈ 1 and R2 > 0.95. In Panels C and D, the sequenced and actual transcript lengths are compared for the remaining 4,741 transcripts in 1994H samples (C) and 2015H samples (D). A much weaker linear correlation holds in both cases, with slope ≠ 1 and R2 ≈ 0.41.

Figure S4. Example of alternative splicing-like coverage pattern. Coverage map of an example transcript in Figure 5, region D (Glyma.15G091400) showing a signal of possible alternative splicing (exon skipping), evident in the abrupt drop in coverage between basepairs 306 and 407 (arrows), followed by a return to initial coverage level. The terminal RNA bases of the skipped exon are 5'-GU and AG-3'. Mean proportional

depth (%) is shown at each position in the transcript (bp) for 1994H (orange line) and 2015H (green line) samples, with standard deviation for each shown in gray.

Figure S5. Coverage maps of transcripts chosen for qPCR assay. Mean proportional depth (%) is shown at each position in the transcript (bp) for 1994H (orange line) and 2015H (green line) samples, with standard deviation for each shown in gray. Transcripts shown in A-C were chosen to represent transcripts that are intact in both harvest years ("intact" transcripts, Fig. 5 Region A), while transcripts shown in D-F were chosen to represent transcripts showing degradation in 1994H samples relative to 2015H samples ("degradation-prone" transcripts, Fig. 5 Region B).

Figure S6. Schematic of transcripts and amplicons. Each bar indicates the full length of the transcript. Each arrow indicates the location and directionality of a primer. Panel A shows intact transcripts chosen from Fig. 5, Region A; Panel B shows degradation-prone transcripts chosen from Fig. 5, Region B. Figure is to scale; scale bar, 100 bp.

Figure S7. Expected qPCR results. A-G model the outcomes of both cDNA synthesis and qPCR for different library preparations performed with intact (A, D, F) or fragmented (B, C, E, G) mRNA using oligo(dT) primers (A, B, C) or random hexamer primers (D, E, F, G) and total RNA (A, C, F, G) or poly(A)-selected mRNA (A, B, D, E) as the template. Blue bar = template mRNA. Purple arrow = primer for cDNA synthesis. Green bar = cDNA. Red arrows = primers for qPCR. Black bars = qPCR amplicons at the same cycle number. When more starting template is present, faster amplification results in a lower Ct value. In H, expected results of ΔCt calculations for each of A-G are shown, facilitating comparison between the results shown in Figure 6.

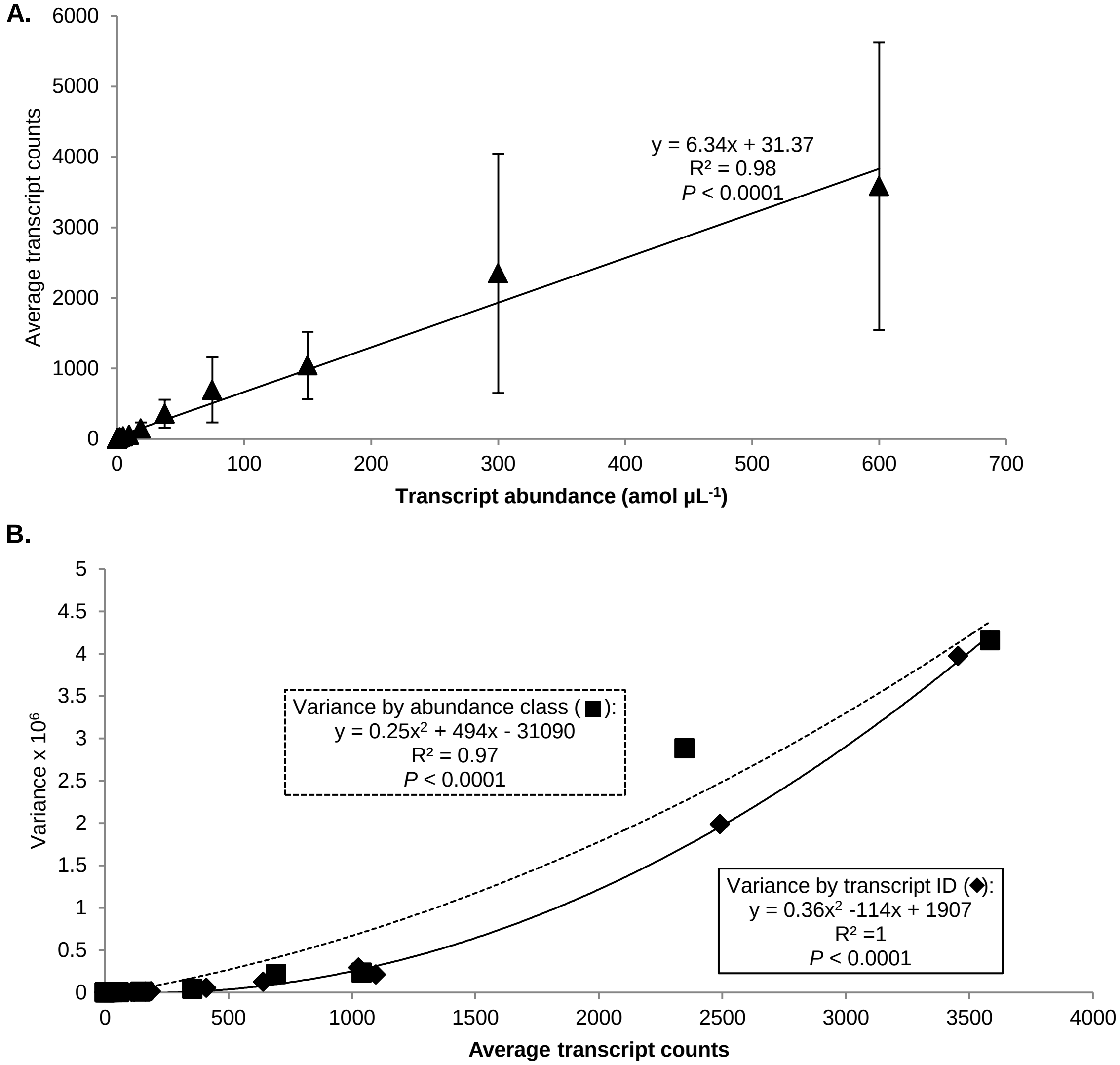

Figure S1. Quality control of sequence data based on ERCC transcripts. A) Average number of counts across all ten samples plotted for each transcript abundance class (22 classes, ranging from 0.0002 to 600 amol µL^{-1} in the dilution used for sequencing). The relationship is strongly linear, indicating that more abundant transcripts are more deeply sequenced, both for ERCC transcripts and soybean transcripts. Error bars show standard deviation. B) Variance in average number of counts across all ten samples for transcript ID (diamonds) or abundance class (squares). The data are strongly related by a quadratic function, indicating that error in the number of counts is more related to the abundance of that transcript than to any variance among the ten samples.

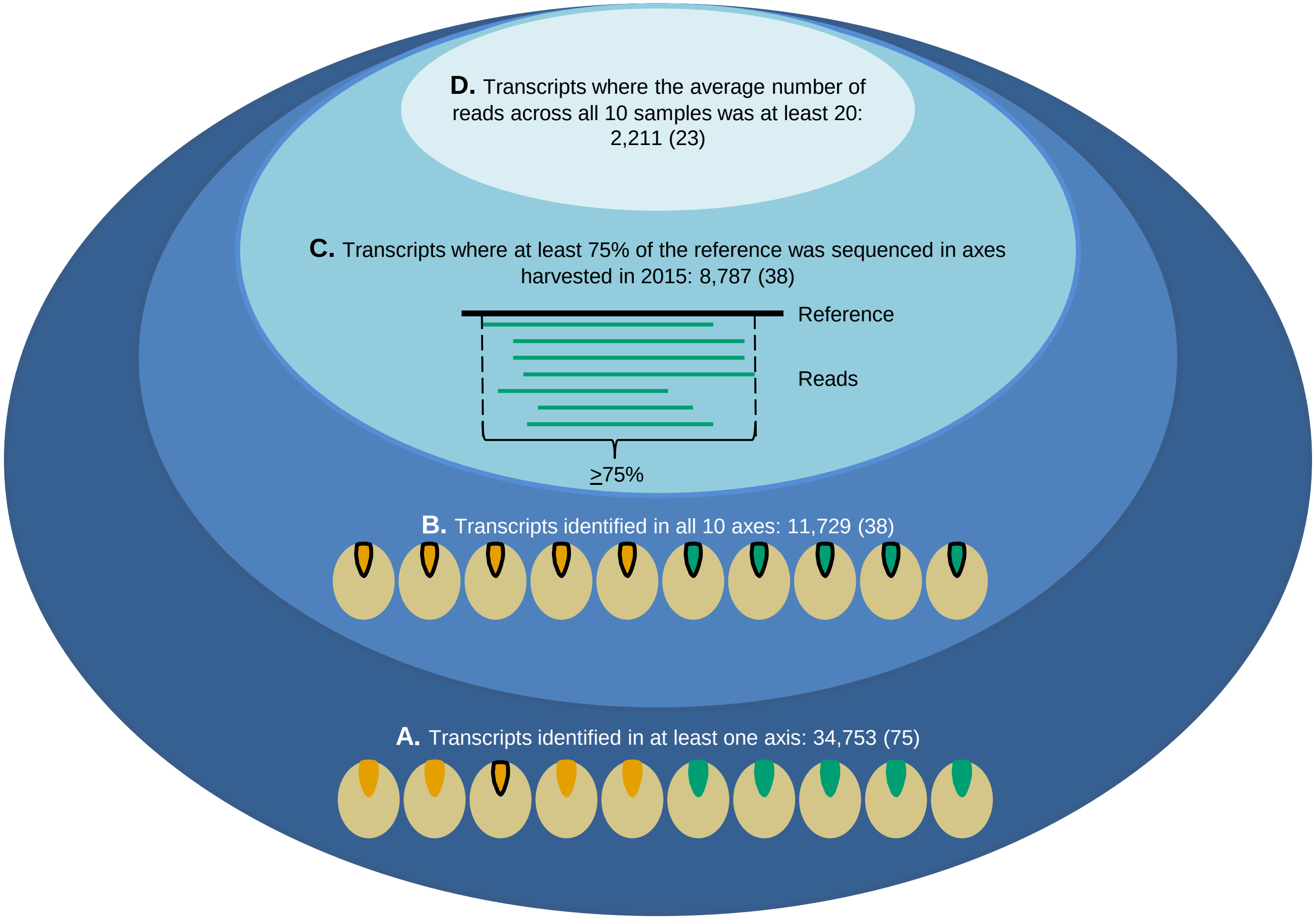

Figure S2. Summary of transcript filtering process. Each panel shows the number of soybean transcripts retained after each step. Embryonic axis color indicates harvest year: 1994 (orange) and 2015 (green). Numbers in parentheses indicate how many transcripts were retained from the 92 transcripts in the ERCC Spike-In mix, which have different sequence lengths (from 273 to 2,022 bp), GC contents (from 30.8 to 52.7%), and abundances (from 0.0003 to 600 amol μL^{-1}, as diluted for sequencing).

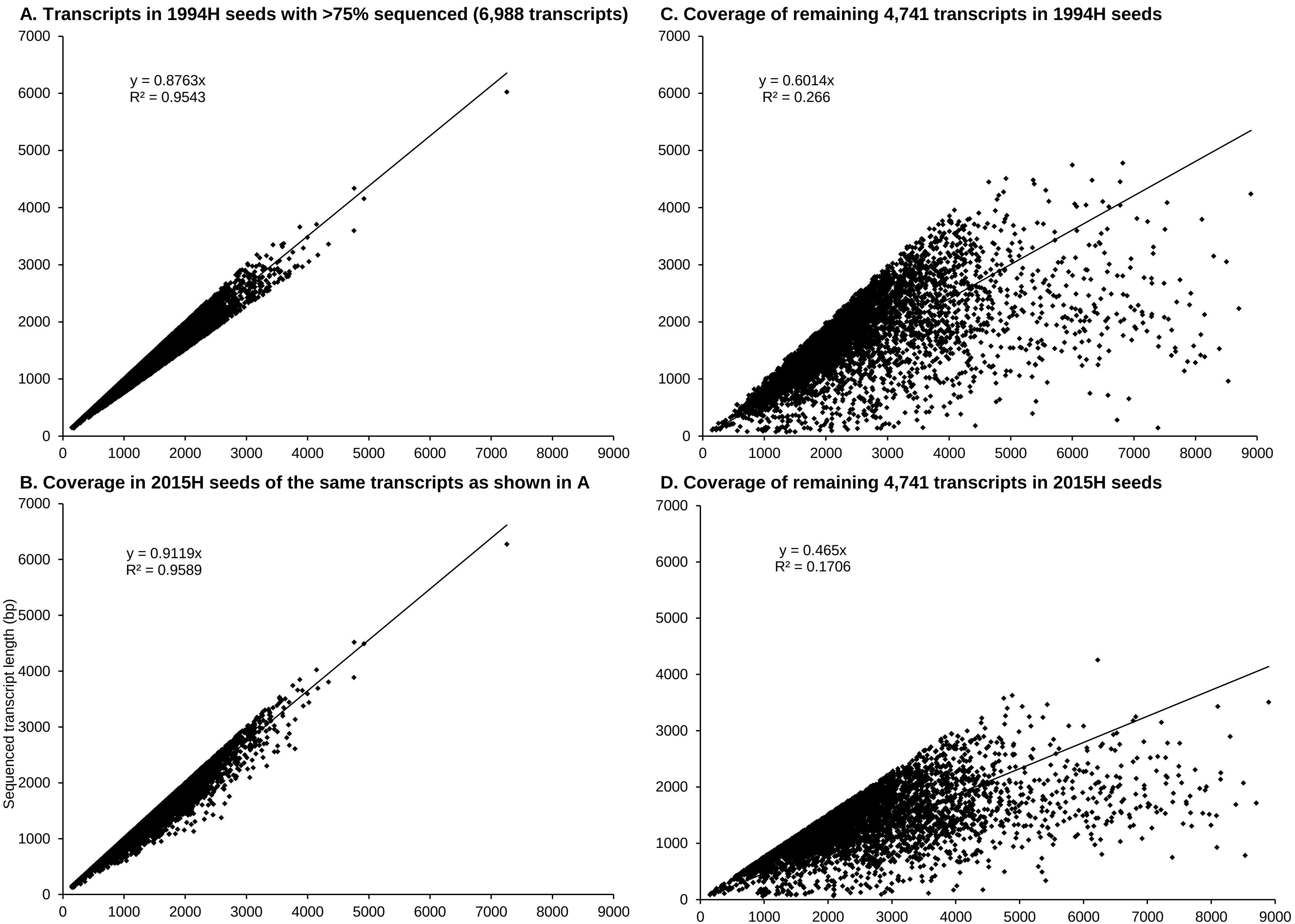
A. Transcripts in 1994H seeds with >75% sequenced (6,988 transcripts)
y = 0.8763x
R² = 0.9543
B. Coverage in 2015H seeds of the same transcripts as shown in A
y = 0.9119x
R² = 0.9589
Sequenced transcript length (bp)
Reference transcript length (bp)
C. Coverage of remaining 4,741 transcripts in 1994H seeds
y = 0.6014x
R² = 0.266
D. Coverage of remaining 4,741 transcripts in 2015H seeds
y = 0.465x
R² = 0.1706
0
1000
2000
3000
4000
5000
6000
7000
8000
9000

Figure S3

Figure S3. Comparison of sequenced transcript length to reference transcript length in the 11,729 transcripts sequenced in all 10 samples. A) Transcripts where the read alignments from 1994H samples covered at least 75% of the reference transcript (6,988 transcripts). B) Sequenced transcript length of the same 6,988 transcripts in 2015H samples. Strong positive linear correlations hold in both cases, with slope ≈ 1 and $R^2 > 0.95$. In Panels C and D, the sequenced and actual transcript lengths are compared for the remaining 4,741 transcripts in 1994H samples (C) and 2015H samples (D). A much weaker linear correlation holds in both cases, with slope ≠ 1 and $R^2 \approx 0.41$.

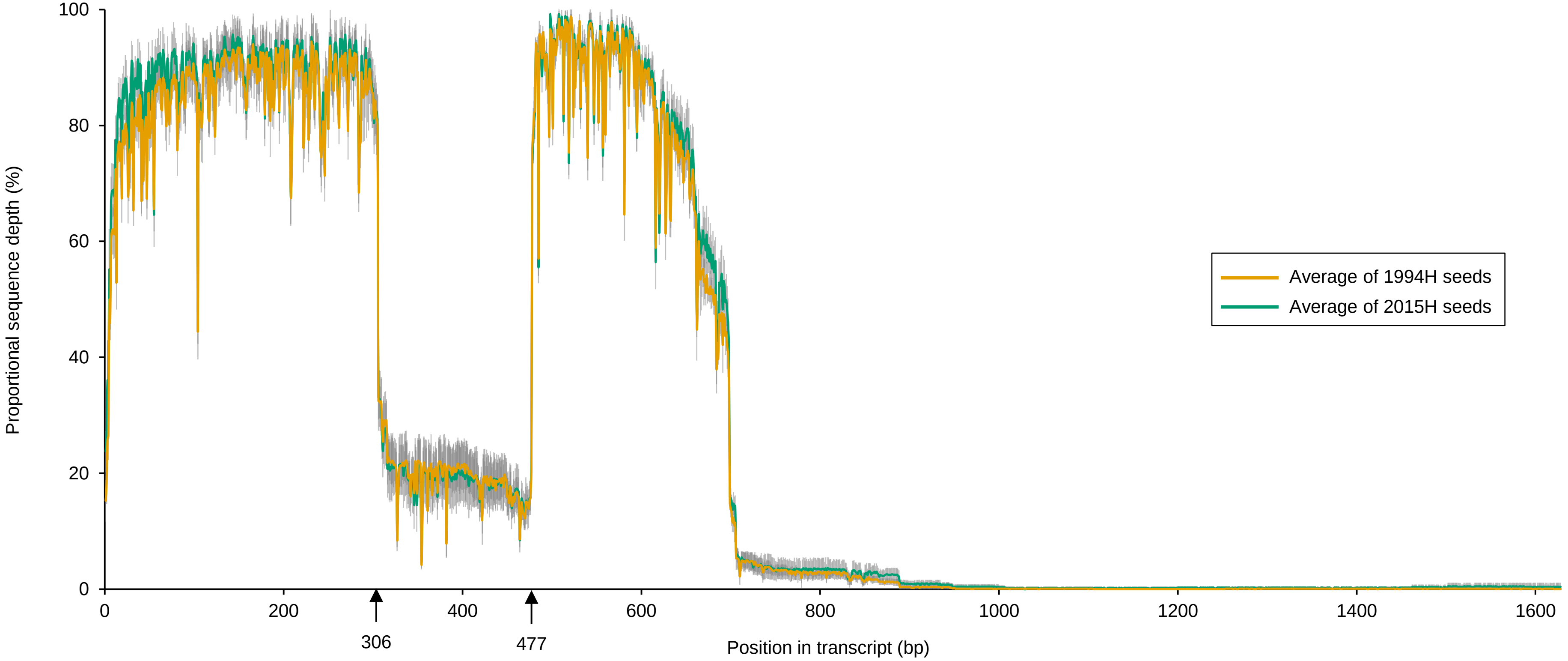

Figure S4. Example of alternative splicing-like coverage pattern. Coverage map of an example transcript in Figure 5, region D (Glyma.15G091400) showing a signal of possible alternative splicing (exon skipping), evident in the abrupt drop in coverage between basepairs 306 and 477 (arrows), followed by a return to initial coverage level. The terminal RNA bases of the skipped exon are 5'-GU and AG-3'. Mean proportional depth (%) is shown at each position in the transcript (bp) for 1994H (orange line) and 2015H (green line) samples, with standard deviation for each shown in gray.

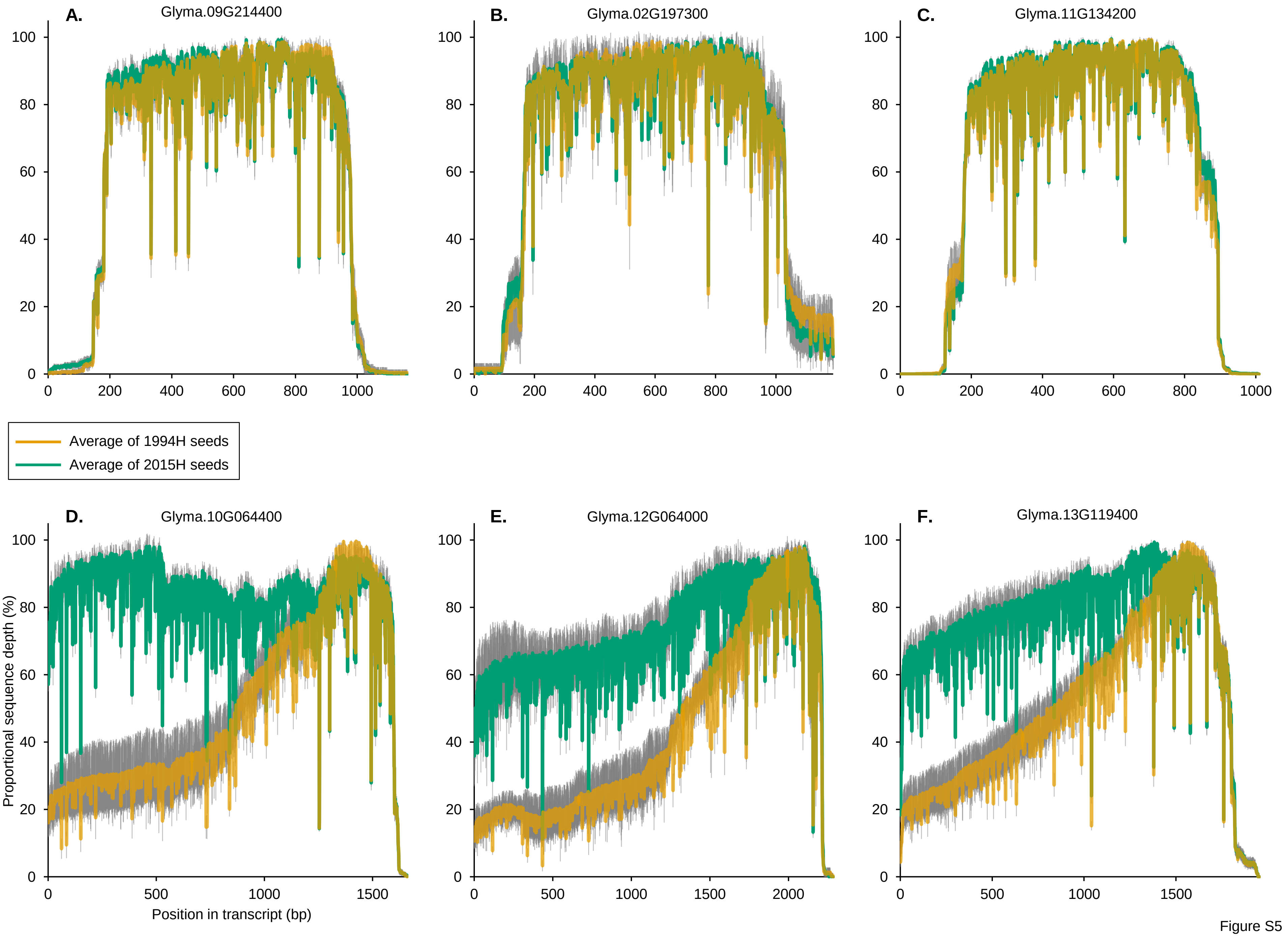
A.
Glyma.09G214400
B.
Glyma.02G197300
C.
Glyma.11G134200
D.
Glyma.10G064400
E.
Glyma.12G064000
F.
Glyma.13G119400
Average of 1994H seeds
Average of 2015H seeds
Proportional sequence depth (%)
Position in transcript (bp)
0
20
40
60
80
100
200
400
600
800
1000
500
1500
2000

Figure S5

Figure S5. Coverage maps of transcripts chosen for qPCR assay. Mean proportional depth (%) is shown at each position in the transcript (bp) for 1994H (orange line) and 2015H (green line) samples, with standard deviation for each shown in gray. Transcripts shown in A-C were chosen to represent transcripts that are intact in both harvest years (“intact” transcripts, Fig. 5 Region A), while transcripts shown in D-F were chosen to represent transcripts showing degradation in 1994H samples relative to 2015H samples (“degradation-prone” transcripts, Fig. 5 Region B).

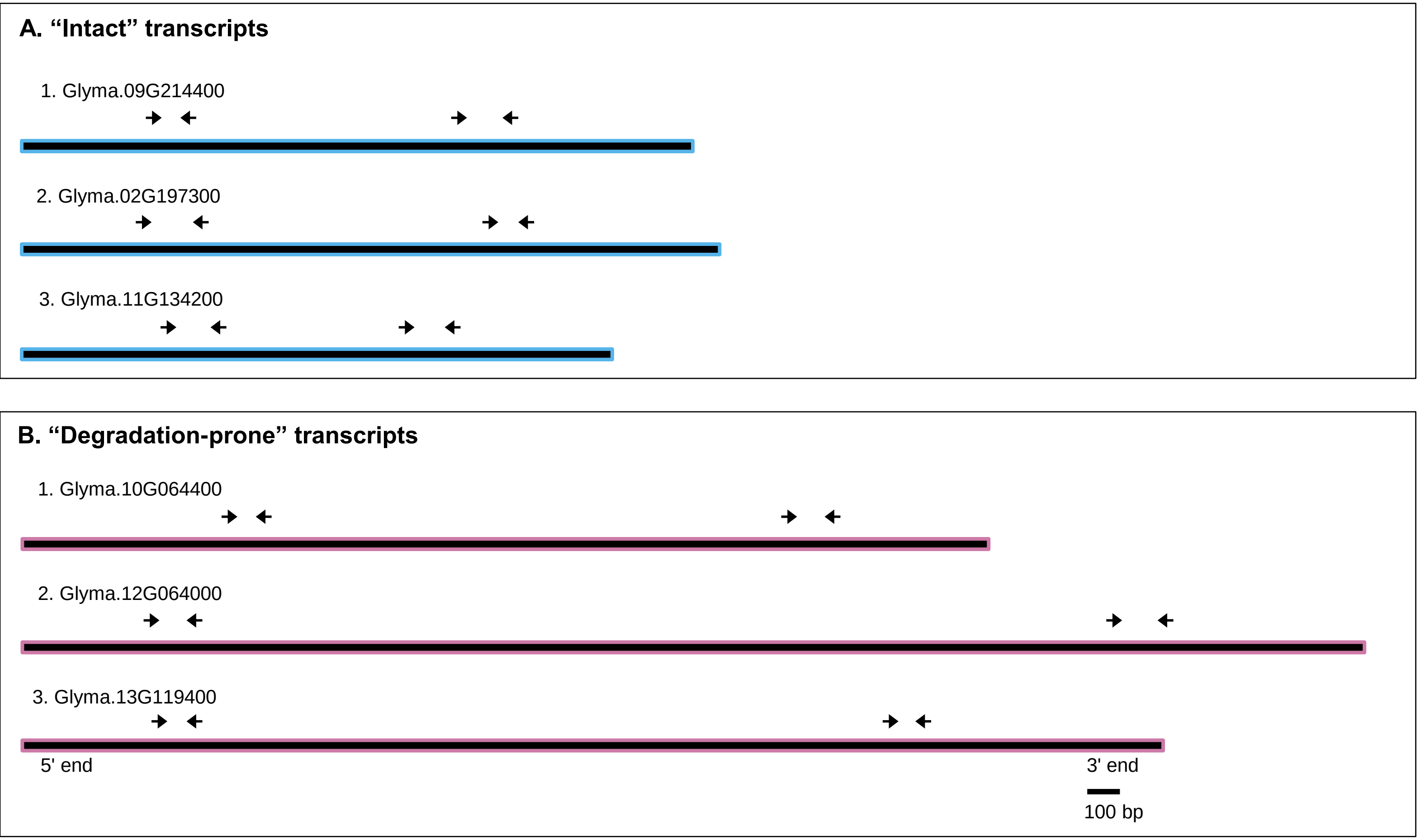

Figure S6. Schematic of transcripts and amplicons. Each bar indicates the full length of the transcript. Each arrow indicates the location and directionality of a primer. Panel A shows intact transcripts chosen from Fig. 5, Region A; Panel B shows degradation-prone transcripts chosen from Fig. 5, Region B. Figure is to scale; scale bar, 100 bp.

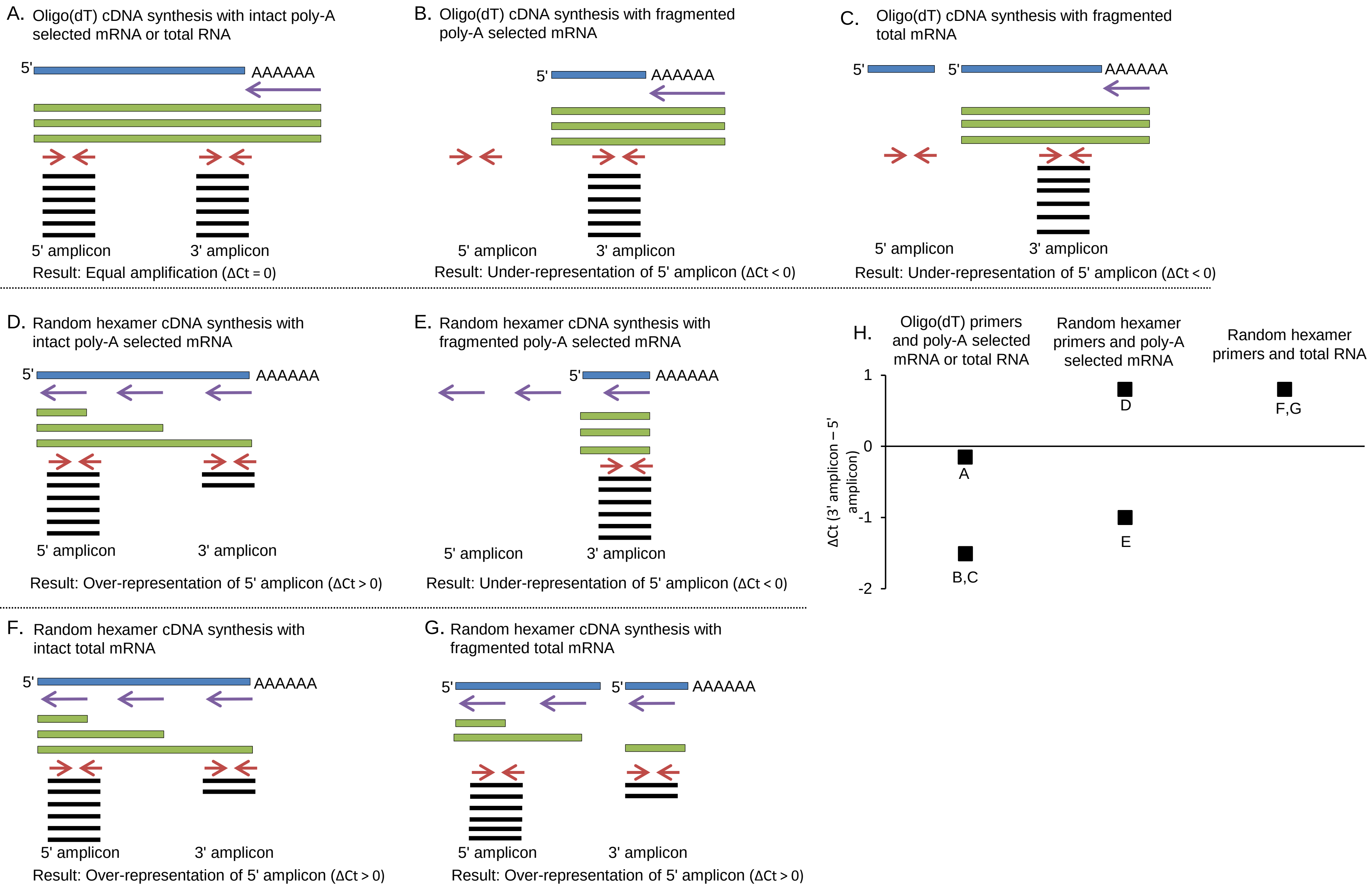
A. Oligo(dT) cDNA synthesis with intact poly-A selected mRNA or total RNA
5'
AAAAAA
5' amplicon
3' amplicon
Result: Equal amplification (ΔCt = 0)
B. Oligo(dT) cDNA synthesis with fragmented poly-A selected mRNA
5'
AAAAAA
5' amplicon
3' amplicon
Result: Under-representation of 5' amplicon (ΔCt < 0)
C. Oligo(dT) cDNA synthesis with fragmented total mRNA
5'
5'
AAAAAA
5' amplicon
3' amplicon
Result: Under-representation of 5' amplicon (ΔCt < 0)
D. Random hexamer cDNA synthesis with intact poly-A selected mRNA
5'
AAAAAA
5' amplicon
3' amplicon
Result: Over-representation of 5' amplicon (ΔCt > 0)
E. Random hexamer cDNA synthesis with fragmented poly-A selected mRNA
5'
AAAAAA
5' amplicon
3' amplicon
Result: Under-representation of 5' amplicon (ΔCt < 0)
H.
Oligo(dT) primers and poly-A selected mRNA or total RNA
Random hexamer primers and poly-A selected mRNA
Random hexamer primers and total RNA
ΔCt (3' amplicon – 5' amplicon)
1
0
-1
-2
A
B,C
D
E
F,G
F. Random hexamer cDNA synthesis with intact total mRNA
5'
AAAAAA
5' amplicon
3' amplicon
Result: Over-representation of 5' amplicon (ΔCt > 0)
G. Random hexamer cDNA synthesis with fragmented total mRNA
5'
5'
AAAAAA
5' amplicon
3' amplicon
Result: Over-representation of 5' amplicon (ΔCt > 0)

Figure S7

Figure S7. Expected qPCR results. A-G model the outcomes of both cDNA synthesis and qPCR for different library preparations performed with intact (A, D, F) or fragmented (B, C, E, G) mRNA using oligo(dT) primers (A, B, C) or random hexamer primers (D, E, F, G) and total RNA (A, C, F, G) or poly(A)-selected mRNA (A, B, D, E) as the template. Blue bar = template mRNA. Purple arrow = primer for cDNA synthesis. Green bar = cDNA. Red arrows = primers for qPCR. Black bars = qPCR amplicons at the same cycle number. When more starting template is present, faster amplification results in a lower Ct value. In H, expected results of ΔCt calculations for each of A-G are shown, facilitating comparison between the results shown in Figure 6.

Table S1. Transcript names and annotations for transcripts exclusive to 1994H or 2015H seeds. Transcripts in A were sequenced in all five 1994H seeds; transcripts in B were sequenced in all five 2015H seeds. Transcript annotations were obtained from Schmutz *et al.* (2010).

	Transcript ID	Transcript annotation
A	Glyma.01G014700	Transcription factor jumonji (jmj) family protein / zinc finger (C5HC2 type) family protein
	Glyma.01G081100	Integrase-type DNA-binding superfamily protein
	Glyma.08G087300	copper-exporting ATPase / responsive-to-antagonist 1 / copper-transporting ATPase (RAN1)
	Glyma.08G227200	calmodulin-domain protein kinase cdpk isoform 2
	Glyma.16G023200	Tetratricopeptide repeat (TPR)-like superfamily protein
	Glyma.18G160300	*No annotation*
	Glyma.20G073500	MUTL protein homolog 1
	Glyma.20G196900	Fe superoxide dismutase 2
B	Glyma.01G034700	TRICHOME BIREFRINGENCE-LIKE 27
	Glyma.03G030900	cytochrome P450, family 83, subfamily B, polypeptide 1
	Glyma.03G078700	plasma membrane intrinsic protein 1;4
	Glyma.10G292900	Nitrilase/cyanide hydratase and apolipoprotein N-acyltransferase family protein
	Glyma.11G184500	S-adenosyl-L-methionine-dependent methyltransferases superfamily protein
	Glyma.11G230000	Phosphoinositide-specific phospholipase C family protein
	Glyma.12G129900	*No annotation*
	Glyma.13G143200	SAUR-like auxin-responsive protein family
	Glyma.19G022700	Disease resistance protein (TIR-NBS-LRR class) family

Table S2. Transcripts longer than 9000 bp sequenced in all 10 samples. %, percent of transcript sequenced. Transcript annotations were obtained from Schmutz *et al.* (2010).

Transcript ID	Transcript annotation	Transcript length (bp)	Sequenced bases (bp)	%
Glyma.07G035600	ATPases; nucleotide binding; ATP binding; nucleoside-triphosphatases; transcription factor binding	16,968	2413	14
Glyma.16G004800	ATPases; nucleotide binding; ATP binding; nucleoside-triphosphatases; transcription factor binding	16,860	3575	21
Glyma.18G275200	Auxin transport protein (BIG)	15,829	2894	18
Glyma.08G252600	Auxin transport protein (BIG)	15,259	3028	20
Glyma.11G039900	Pleckstrin homology (PH) domain-containing protein	13,842	2254	16
Glyma.11G077000	Phosphatidylinositol 3- and 4-kinase family protein with FAT domain	12,488	2645	21
Glyma.01G166400	Phosphatidylinositol 3- and 4-kinase family protein with FAT domain	12,365	1808	15
Glyma.11G123500	Target of rapamycin	12,313	2337	19
Glyma.07G252100	P-loop containing nucleoside triphosphate hydrolases superfamily protein	12,033	3899	32
Glyma.08G087600	Ubiquitin-protein ligase 1	12,005	2544	21
Glyma.05G133100	Ubiquitin-protein ligase 1	11,916	3987	33
Glyma.02G216000	Ubiquitin-protein ligase 1	11,528	1993	17
Glyma.17G022300	P-loop containing nucleoside triphosphate hydrolases superfamily protein	11,159	2602	23
Glyma.06G098700	Ubiquitin-protein ligase 1	10,917	2053	19
Glyma.14G142900	ENTH/VHS/GAT family protein	10,869	2097	19
Glyma.04G096100	Binding	10,658	2871	27
Glyma.14G143000	ENTH/VHS/GAT family protein	10,327	1324	13
Glyma.05G185300	*No annotation*	10,272	2012	20
Glyma.16G144200	Protein of unknown function (DUF1162)	9624	1316	14

Glyma.17G197200	*No annotation*	9457	2682	28
Glyma.14G135900	*No annotation*	9194	3459	38
Glyma.13G174600	Protein of unknown function (DUF1162)	9071	2195	24
Glyma.06G098300	Golgi-body localization protein domain; RNA pol II promoter Fmp27 protein domain	9025	1584	17

Table S3. Transcripts with unexpected decay profiles given their length. A) Transcripts found in Figure 5, region A. B) Transcripts found in Figure 5, region B. RD_{2015}, degradation of 2015H samples relative to an intact transcript; RD_{Δ}, degradation of 1994H samples relative to 2015H samples. Transcript annotations were obtained from Schmutz *et al.* (2010).

	Transcript ID	Length (bp)	RD_{2015}	RD_{Δ}	Transcript annotation
A	Glyma.08G346500	1236	0.11	0.00	Ribosomal protein S8e family protein
	Glyma.12G024700	1224	0.11	0.01	Rotamase CYP 3 protein
	Glyma.13G116300	1210	0.10	0.02	Transmembrane protein 14C
B	Glyma.02G049300	1112	0.15	0.16	Ribosomal protein L2 family
	Glyma.10G257200	1010	0.13	0.15	*No annotation*
	Glyma.13G064900	1188	0.19	0.17	Voltage dependent anion channel 1
	Glyma.13G237700	1021	0.20	0.24	Late embryogenesis abundant protein
	Glyma.15G191100	737	0.17	0.15	*No annotation*
	Glyma.16G112800	2572	0.19	0.23	RNA polymerase I specific transcription initiation factor RRN3 protein

Table S4. GC content in each region of Figure 5. GC contents show mean ± standard error for transcripts in that region. Different letters indicate $P < 0.05$ (Tukey's HSD).

Region	Transcript degradation	Number of transcripts	GC content (%)
A	intact in 2015H and 1994H	198	42 ± 0.003[a]
B	intact in 2015H, degraded in 1994H	114	44 ± 0.003[c]
C	degraded in 2015H, more degraded in 1994H	99	43 ± 0.003[b,c]
D	degraded in 2015H and 1994H	104	42 ± 0.005[a]
none	variable	1719	43 ± 0.0008[b]

Table S5. Over-represented GO terms for region C in the molecular function GO domain. Over-representation was determined by the PANTHER Overrepresentation test using a Bonferroni correction for multiple testing, with a cutoff of $P < 0.05$. Ref. = number of the 1821 annotated curated transcripts with this annotation; Reg. C = number of the 91 annotated transcripts in region C with this annotation; FE = Fold Enrichment, tri-P = triphosphate. Annotations are listed hierarchically, with the most specific (child) annotations in bold, and parent annotations successively indented.

Transcript annotation	GO ID	Ref. (1821)	Reg. C (91)	FE	P-value
**ATP binding**	**GO:0005524**	**113**	**22**	**3.90**	**9.78E-06**
adenyl ribonucleotide binding	GO:0032559	113	22	3.90	9.78E-06
adenyl nucleotide binding	GO:0030554	114	22	3.86	1.14E-05
purine nucleotide binding	GO:0017076	142	26	3.66	1.36E-06
nucleotide binding	GO:0000166	159	28	3.52	6.72E-07
nucleoside phosphate binding	GO:1901265	159	28	3.52	6.72E-07
heterocyclic compound binding	GO:1901363	556	47	1.69	7.25E-03
binding	GO:0005488	727	55	1.51	2.08E-02
organic cyclic compound binding	GO:0097159	556	47	1.69	7.25E-03
small molecule binding	GO:0036094	172	29	3.37	8.47E-07
purine ribonucleotide binding	GO:0032555	141	26	3.69	1.17E-06
ribonucleotide binding	GO:0032553	142	26	3.66	1.36E-06
carbohydrate derivative binding	GO:0097367	143	26	3.64	1.57E-06
purine ribonucleoside tri-P binding	GO:0035639	141	26	3.69	1.17E-06
anion binding	GO:0043168	161	28	3.48	8.88E-07
ion binding	GO:0043167	289	34	2.35	1.90E-04
**catalytic activity**	**GO:0003824**	**420**	**38**	**1.81**	**1.93E-02**

Table S6. Germination rate and RNA quality of seed lots used for RNA oxidation measurements. Germination rates were measured in 2017. RNA data show the mean ± SD (n = 5) of RNA extracted from six pooled embryonic axes. RNA yield, µg RNA mg^{-1} sample tissue. Different letters indicate $P < 0.05$ (Tukey's HSD).

Harvest year	Germination %	RNA yield	RIN
2015	100	5.81 ± 1.58[a]	7.72 ± 0.33[a]
1999	90	5.16 ± 1.21[a]	7.22 ± 0.19[ab]
1995	28	3.43 ± 0.91[a]	6.74 ± 0.05[bc]
1989	0	2.64 ± 0.87[a]	5.96 ± 0.18[c]

Table S7. Genes and amplicons chosen for qPCR. Transcripts with similar lengths were selected to represent two regions in Figure 5, region A (“intact”) and region B (“degradation-prone”). MinION sequence data were used to ensure the 5' and 3' amplicons fell within the sequenced portion of the transcript in 2015H samples. %, percent of transcript length to first base of amplicon.

Transcript category	Transcript ID	Length (bp)	Location of 5' amplicon (bp)	%	5' amplicon length (bp)	Location of 3' amplicon (bp)	%	3' amplicon length (bp)
Intact	Glyma.09g214400	1161	215-305	18.5	90	749-848	64.5	99
	Glyma.02g197300	1190	204-320	17.1	116	789-874	66.3	85
	Glyma.11g134200	1009	241-345	23.9	104	655-755	64.9	100
Degradation-prone	Glyma.10g064400	1660	325-420	19.6	95	1305-1398	78.6	93
	Glyma.12g064000	2285	192-294	8.4	102	1861-1961	81.4	100
	Glyma.13g119400	1953	218-313	11.2	95	1468-1554	75.2	86

Table S8. Primer sequences for each qPCR amplicon. (5'/3'), amplicon occurs at the 5' or 3' end of the transcript.

Transcript category	Sequence accession number	(5'/3')	Forward primer (5' → 3')	Reverse primer (5' → 3')
Intact	Glyma.09g214400	5'	TTATCCCAAACGGACACTTCC	GCCAATCGTCTCCTTGTCTT
		3'	GCACAAACAAACGCCATCA	GACTACAAACGTAAGACTTCTAAAGG
	Glyma.02g197300	5'	ATAATGATCGACGGAGGAAGC	CGTGAGGAGGCTCTTGAAAT
		3'	ATTAACGGCGGCTTTCACCT	TTCGCCGGCCGTGTAATC
	Glyma.11g134200	5'	GATGCCAACGGCTCCAA	TCCGAATCCGAATTCCAAGC
		3'	GACTGAACCCAGTGACCAAA	AACAGAGCAGGGTAGGGTAT
Degradation-prone	Glyma.10g064400	5'	CGTACAGTACCAGAGAGGTTATTC	GTGACTTTGAGGCTGATTTGTC
		3'	GCAATTGCGGAAACACTGAA	CATTGACGACACCCTCTTCTT
	Glyma.12g064000	5'	CATAGCCAACGATCAGGGTAAT	GTTCATAGCGACCTGGTTCTT
		3'	GATGGAAACCAACTTGCTGAAG	CCAGCTCCTTGGTACATCTTT
	Glyma.13g119400	5'	CCAAAGAGGAAGGTCACGATTT	CGGCTTCTTGTCCCTCTTTAC
		3'	GACATAGCAAAGGCTGCAAAG	CAATTCTCAAAGCTCGGCATC

Table S9. Ct values and efficiency calculations for each qPCR primer set. cDNA synthesized from a single 2015H embryonic axis was diluted in a 2x dilution series; RT-qPCR was performed to determine the Ct values for each dilution. Efficiency was calculated as $10^{-(1/slope)}$, where the slope was derived from the linear regression of the Ct values for the dilution series for that transcript. E, efficiency.

Transcript category	Primer set	Dilution (log of dilution factor) 0	-0.30	-0.60	-0.90	-1.20	-1.50	-1.81	-2.11	Linear regression	R^2	E
Intact	Glyma.09g214400 5' amplicon	25.66	26.28	27.29	28.47	29.29	30.27	31.10	33.16	y = -3.4153x + 25.342	0.98	1.96
	Glyma.09g214400 3' amplicon	24.70	25.38	26.36	27.30	28.22	29.39	30.05	31.64	y = -3.2405x + 24.466	0.99	2.04
	Glyma.02g197300 5' amplicon	26.13	26.88	27.83	28.91	29.92	31.12	32.21	33.42	y = -3.5023x + 25.863	1.00	1.93
	Glyma.02g197300 3' amplicon	25.70	26.32	27.31	28.23	29.35	29.75	31.03	32.29	y = -3.0894x + 25.493	0.99	2.11
	Glyma.11g134200 5' amplicon	24.19	24.84	25.59	26.69	27.60	28.50	29.44	30.58	y = -3.0597x + 23.955	1.00	2.12
	Glyma.11g134200 3' amplicon	23.01	23.97	24.68	25.56	26.60	27.39	28.28	29.60	y = -3.0392x + 22.934	1.00	2.13
Degradation-prone	Glyma.10g064400 5' amplicon	20.79	21.59	22.43	24.20	24.37	25.51	26.42	27.61	y = -3.2152x + 20.728	0.99	2.05
	Glyma.10g064400 3' amplicon	19.74	20.47	21.35	22.34	23.31	24.38	25.41	26.51	y = -3.2488x + 19.516	1.00	2.03
	Glyma.12g064000 5' amplicon	24.05	24.82	25.63	26.82	27.50	28.69	29.67	30.76	y = -3.2065x + 23.864	1.00	2.05
	Glyma.12g064000 3' amplicon	23.40	24.32	25.14	26.14	27.23	28.13	29.26	30.10	y = -3.2294x + 23.313	1.00	2.04
	Glyma.13g119400 5' amplicon	21.46	22.44	23.37	24.36	25.25	26.18	27.39	28.14	y = -3.1966x + 21.456	1.00	2.06
	Glyma.13g119400 3' amplicon	19.81	20.77	21.60	22.62	23.56	24.73	25.54	26.68	y = -3.2535x + 19.736	1.00	2.03

Protocol S1. Detailed description of Oxford Nanopore cDNA synthesis and sequencing library preparation.

Synthesis of cDNA for sequencing

Reagents were supplied by Invitrogen unless otherwise noted. Reverse transcription reactions consisted of 17 ng of template RNA, 1 µL of a 1:50 dilution of ERCC RNA Spike-In Mix (Ambion), 2 µL of 1 µM poly(T)-VN primer (5' phosphate/ACTTGCCTGTCGCTCTATCTTCT_{20}VN, Integrated DNA Technologies), 1 µL of 10 mM dNTPs, and nuclease-free water up to a final reaction volume of 11 µL. Reactions incubated at 65 °C for 5 min were snap-cooled in a pre-chilled metal tube rack for 1 min, followed by addition of 4 µL of RT buffer, 1 µL of 100 mM DTT, 40 U of RNAse OUT, and 2 µL of 10 µM strand-switching primer (TTTCTGTTGGTGCTGATATTGCTGCCATTACGGCCmGmGmG, Integrated DNA Technologies). Reactions were then incubated at 42 °C for 2 min, followed by the addition of 200 U of SuperScript® IV Reverse Transcriptase. Reverse transcription occurred at 50 °C for 10 min, followed by strand-switching at 42 °C for 10 min and heat inactivation at 80 °C for 10 min. The resulting cDNA was purified with 14 µL of Agencourt AMPure XP beads (Beckman), washing three times with freshly prepared 80% ethanol and eluting in 21 µL of nuclease-free water after a 10 min incubation at 22 °C on a Hula Mixer (Invitrogen).

Library barcoding, pooling, and sequencing prep

Unique barcodes (Oxford Nanopore Technologies, EXP-PBC001) were added to each sample during cDNA amplification according to the Oxford Nanopore recommended protocol. The barcoding reaction consisted of 20 µL reverse transcription reaction, 2 µL of barcode primer, 8 µL of nuclease-free water, and 30 µL of LongAmp Taq 2x MasterMix (New England Biolabs). Samples underwent PCR as follows: initial denaturation at 95 °C for 3 min, 18 cycles of 95 °C for 15s, 62 °C for 15s, 65 °C for 100s, final extension at 65 °C for 10 min. Amplified cDNA was purified with 108 µL of Agencourt AMPure XP beads, washing twice with freshly prepared 80% ethanol and eluting in 22 µL of nuclease-free water. Samples were combined into two pools, each

containing 1 µg of cDNA in 45 µL (200 ng of cDNA from each of five samples). The first pool included two 1994H and three 2015H samples, while the second pool included three 1994H and two 2015H samples.

Each pooled cDNA library was prepared for sequencing with the NEBNext End repair/dA-tailing module (New England Biolabs) in a 60 µL reaction that included 5 µL of a control DNA strand supplied by Oxford Nanopore Technologies, 7 µL of Ultra II End-prep reaction buffer, and 3 µL of Ultra II End-prep enzyme mix. Reactions were incubated for 5 min at 20 °C, followed by 5 min at 65 °C. Enzymes were removed by binding the cDNA to 60 µL Agencourt AMPure XP beads, washing twice with freshly prepared 80% ethanol, and then eluting in 31 µL of nuclease-free water after a 10 min incubation at 22 °C. Oxford Nanopore Technologies adapters were ligated to 10 µL of end-prepped cDNA in a 100 µL reaction containing 20 µL of adapter mix AMX1D (Oxford Nanopore Technologies) and 50 µL of Blunt/TA Ligase Master Mix (New England Biolabs). The reaction was then incubated for 10 min at 22 °C. Libraries were purified once again using 40 µL of Agencourt AMPure XP beads but washed twice with 140 µL of Adapter Bead Binding buffer (Oxford Nanopore Technologies), and eluted in 15 µL of Elution Buffer (Oxford Nanopore Technologies) after 10 min incubation at 22 °C on a Hula Mixer. Libraries were mixed with 35 µL of Running Buffer with Fuel Mix (RBF, Oxford Nanopore Technologies) and 25.5 µL of Library Loading Beads (Oxford Nanopore Technologies). Each library was sequenced on a MinION SpotON Flow Cell MK I (R9.4) (Oxford Nanopore Technologies). Flow cells were primed by loading 800 µL of priming mix (480 µL of RBF and 520 µL of nuclease-free water) into the priming port. After 5 min of priming, the SpotON sample port was opened and 200 µL of priming mix was loaded into the priming port, followed immediately by 75 µL of thoroughly mixed library added dropwise to the sample port. The SpotON sample port was closed and sequencing was initiated with the sequencing script "NC_48Hr_Sequencing_Run_FLO-MIN106_SQK-LSK108" in MinKNOW 1.4.2.
